# Integrated distribution modeling resolves asynchrony between bat population impacts and occupancy trends through latent abundance

**DOI:** 10.1038/s42003-025-08238-x

**Published:** 2025-05-30

**Authors:** Bradley J. Udell, Christian Stratton, Kathryn M. Irvine, Bethany Rose Straw, Jonathan D. Reichard, Sarah Gaulke, Jeremy. T. H. Coleman, Frank C. Tousley, Andrea N. Schuhmann, Richard D. Inman, Melinda Turner, Sarah Nystrom, Brian E. Reichert

**Affiliations:** 1 United States Geological Survey, Fort Collins Science Center, Fort Collins, CO USA; 2 Department of Mathematical Sciences, Montana State University, Bozeman, MT USA; 3 United States Geological Survey, Northern Rocky Mountain Science Center, Bozeman, MT USA; 4 United States Fish and Wildlife Service, Ecological Services, Hadley, MA USA; 5 Colorado Cooperative Fish and Wildlife Research Unit Department of Fish, Wildlife, and Conservation Biology Colorado State University Fort Collins, Fort Collins, CO USA; 6 Colorado Natural Heritage Program Colorado State University Fort Collins, Fort Collins, CO USA; 7 United States Fish and Wildlife Service, Ecological Services, State College, PA USA

**Keywords:** Ecological modelling, Population dynamics, Macroecology, Conservation biology, Animal migration

## Abstract

Monitoring populations is challenging for cryptic species with seasonal life cycles, where data from multiple field techniques are commonly collected and analyzed as multiple lines of evidence. Data integration can provide comprehensive inferences while improving accuracy, precision, and scope but faces challenges in modeling misaligned resolutions and observational uncertainties. We developed a multi-scale, integrated species distribution model (MS-iSDM) for North American bats to combine data across monitoring types and seasons using joint likelihood methods, observational models with false-negatives and false-positives, and seasonal migratory connectivity. We applied this model to 11 years of data for an imperiled bat species (tricolored bat, *Perimyotis subflavus*). Relative abundance and occupancy were linked with multi-scale predictors, revealing clear patterns of population declines, but with important differences in spatial trends (abundance: corresponded with white-nose syndrome impacts, occupancy: at the range periphery) and overall severity (abundance: -74.8%, 95% CRI: -79.7% to -69.3%; occupancy: -35.5%, 95% CRI: -41.1% to -30.2%). The asynchrony between occupancy trends and population impacts was explained as an emergent pattern of spatiotemporal variation in abundance in the integrated distribution model. Compared to multiple lines of evidence, the integrated model provided consensus-estimates, increased precision and spatiotemporal scope, and strengthened evidence of population declines.

## Abstract (web summary)

Integrated species distribution modeling across monitoring types, spatial scales, and seasons resolves asynchrony in population trends for an imperiled bat species (Perimyotis subflavus) and strengthens evidence of declines linked to wildlife disease.

## Introduction

Understanding where organisms occur and in what local densities, as well as how and why species distributions change in space and time, are central themes in ecology^1–3^, biogeography^2,3^, and conservation biology^4^. Especially of interest is how species’ occupancy and abundance distributions relate to environmental conditions^1–3^, population processes^1–3^, stressors^4^, management actions^4^, or with one-another^1–3^. Conservation monitoring programs collect and analyze ecological data to address these questions and to inform where and in what intensities limited management actions might be targeted to achieve conservation goals^4–8^. However, despite advancements in collaborative monitoring programs at macro scales^9,10^ and in statistical methods to estimate occupancy and abundance distributions^11,12^, extracting simple and comprehensive answers to such questions remains challenging^12^.

Large-scale monitoring often requires that several field techniques be deployed across different spatial and temporal resolutions, especially for species with cryptic behaviors, seasonal life cycles, migratory behavior, and continental distributions^6,8,13^. Data collected from different field techniques and seasons may be prone to different sources and magnitudes of observation bias (false-negatives, false-positives, differential effort) and be best suited to inform different population metrics (occupancy vs abundance). For example, bats in North America exemplify these challenges^6,8^. The North American Bat Monitoring Program^8,10^ (NABat) was established in 2015 to address a historical lack of coordinated population monitoring efforts for most species of North American bats^6^ in the wake of white-nose syndrome (WNS), which has caused devastating population declines of hibernating bats^14,15^. NABat uses information from multiple monitoring methodologies to gain a richer understanding of population status and trends^6,8^, including acoustics (deployed at stationary point locations and along mobile transects) and live-capture data in the summer maternity season. In the winter, counts of individuals within hibernating bat colonies are also obtained. From these streams of monitoring data, population status and trend inferences are based on (1) species occupancy in the summer^16,17^, (2) relative abundance in the summer^18^, or (3) relative abundance in the winter^14,19^. Thus, population inferences to date have been mostly confined to ‘multiple lines of evidence’ which are analyzed independently as opposed to ‘combined inference’ under a single framework.

Data integration provides a promising avenue for combining multiple monitoring efforts into a single inferential framework—integration can increase accuracy, precision, and spatiotemporal scope of inference relative to independent analyses^11,12^. Importantly, as all data sets infer shared population parameters, data integration can produce comprehensive estimates of species distributions and trends over time while appropriately weighting informational quantity and quality^11^. However, several analytical challenges must be overcome^12^ including modeling response data with misaligned spatial and temporal resolutions and potentially different observational biases and uncertainties^20^. Combining occupancy metrics (less sensitive to population change) with abundance-based metrics presents an additional challenge given the typical non-linear and saturating relationship between them^21^. This relationship precludes simple averaging and requires mechanistic statistical approaches for integration^21–24^.

This latter challenge is also present in post-hoc comparisons of occupancy and abundance-based metrics that have been analyzed independently, where intuition alone may be insufficient. For example, one imperiled bat species (tricolored bat, *Perimyotis subflavus*) has experienced catastrophic declines in abundance due to white-nose syndrome (as reflected in trends of abundance-based^18,19^ and activity-based^25^ metrics) but remains widespread in summertime occupancy throughout much of its range^17,25^. This asynchrony in observed trends between occupancy-based and abundance-based metrics could lead to ambiguity or confusion when informing conservation management^25^. Integrated species distribution models (iSDMs)^22–24^, which simultaneously model occupancy and abundance via a shared latent abundance process, provide an opportunity to unify inferences across population metric despite apparently disparate patterns. In particular, iSDMs deterministically link occupancy and abundance distributions via a shared spatial point pattern process and probability of zero given an assumed spatial mesh^24^.

Specifying a useful relationship in space and time between seasonally monitored populations presents another challenge of data integration. For example, most bat species in North America resemble metapopulations with seasonal dynamics, occurring as discrete seasonal colonies (e.g., winter hibernacula, summer maternity, summer bachelor), which are governed by local population dynamics (births, survival), and linked within seasons (by dispersal) and between seasons (by migration). Population impacts from WNS primarily occur in the winter^14^ but also manifest in summer populations which are connected via seasonal migration. Metapopulation^26^ and landscape connectivity theory^27,28^ predict functional relationships to quantify expected animal movement and dispersal in space and time as measures of ‘potential’ population connectivity. For example, a seasonal migratory connectivity approach^18^ can predict the spatiotemporal distribution of expected migrants in the summer as a function of the species migration behavior and the spatiotemporal distribution of winter populations. Then, it can be included as a covariate to predict summer abundance and occupancy in an integrated species distribution model to quantify the potential linkage between winter and summer populations in space and time.

**Fig. 1:**
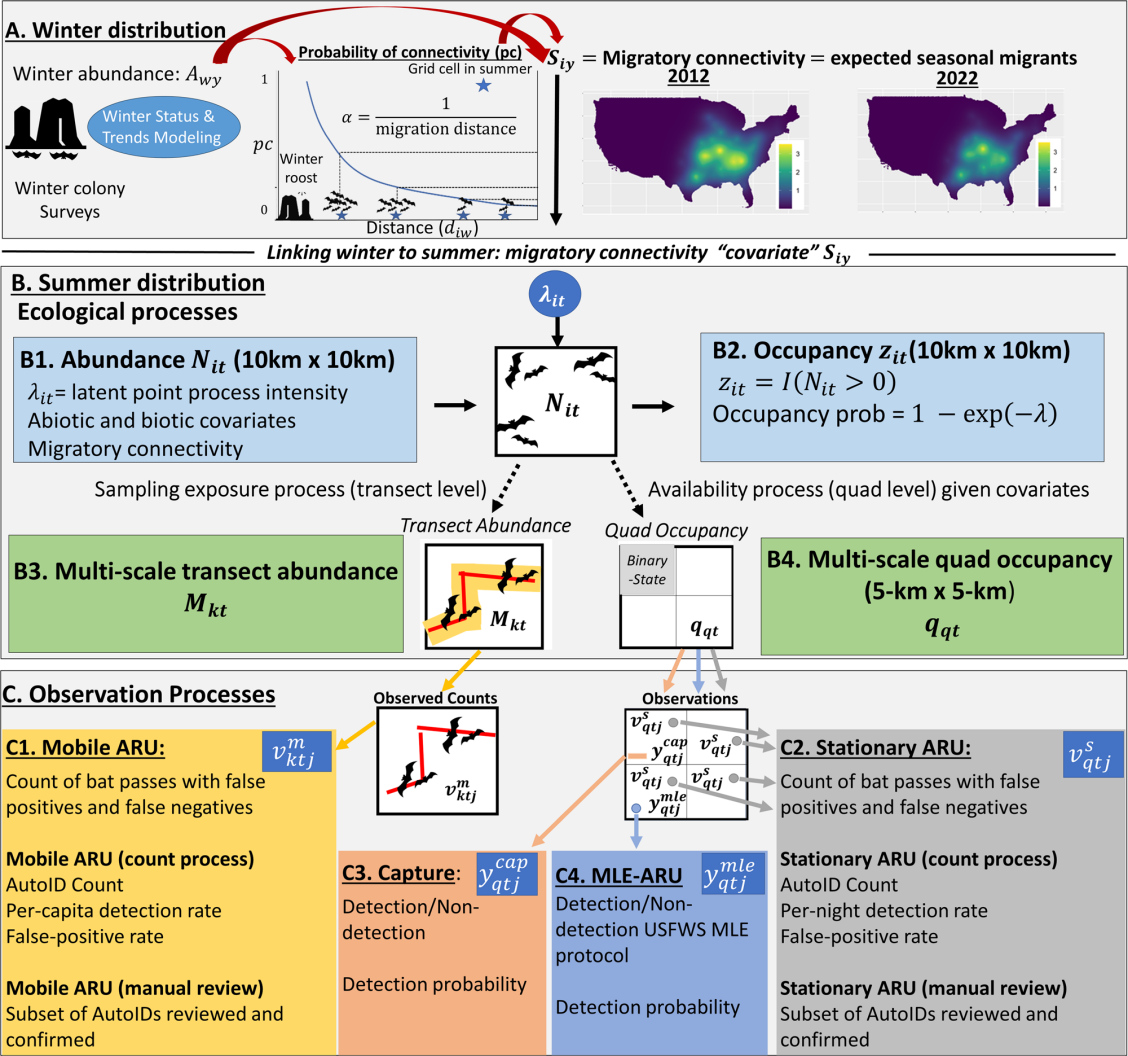
A multi-scale integrated species distribution model (MS-iSDM) for the tricolored bat (*Perimyotis subflavus*) illustrating the connections between winter populations, summer populations, and data integration via observation models. Let *i* indicate NABat grid cells (10-km x 10-km), *q* indicate quadrants (5-km x 5-km), *k* indicate mobile transect routes, *y* indicate year, *t* indicate time periods (years $\times$ within-summer-season), and *w* indicate winter colonies. **A** Estimates of winter colony abundance each year $({A}_{{wy}})$ are provided by previous NABat analyses. The expected number of summer migrants ${S}_{{iy}}$ to each grid cell each year is calculated as abundance weighted dispersal flux (given ${A}_{{wy}}$, distances between locations, and migration kernel) and used as covariate for summer abundance. **B** Summer abundance ${N}_{{it}}$ (and occupancy state ${z}_{{it}}$) for each grid cell and time period are estimated as a function of spatiotemporal covariates and a temporally autocorrelated annual intercept. ${N}_{{it}}$ and ${z}_{{it}}$ are assumed open between early and late summer seasons due to a mid-summer birth-pulse, where ${\beta }_{{post}}$ is the post-volancy effect on abundance. Multi-scale abundance ${M}_{{kt}}$ is modeled for each transect and time period conditional on ${N}_{{it}}$ and the sampling exposure rate (function of transect length). Multi-scale occupancy ${q}_{{qt}}$ is modeled for each quadrant and time period conditional on ${z}_{{it}}$ and the probability of local availability (a function of covariates). C1. For mobile ARUs (automated recording units), the count of detections each night ${v}_{{ktj}}$ comes from a Poisson process given ${M}_{{kt}}$, the per-individual detection rate ${\delta }_{{ktj}}^{m}$ (a function of covariates and random effects), and the false-positive rate ${\omega }_{{ktj}}^{m}$ (with observation-level random effects by data contributor). A hypergeometric observation model was assumed for the manual review process, for subsets of reviewed ${n}_{{ktj}}^{m}$ and confirmed ${k}_{{ktj}}^{m}$ recordings. C2: For stationary ARUs, a Poisson count-detection process was assumed conditional on ${q}_{{qt}}$, detection rates ${\delta }_{{qtj}}^{s}$, and false positive rates (similar to C1). The same manual review observation model was assumed as C1. C3 and C4: a traditional multi-scale occupancy model detection process is assumed for live-capture data (C3) conditional on ${q}_{{qt}}$ and the detection probability and (C4) also for maximum likelihood estimator ‘MLE’ style stationary acoustic records (assumed no false-positive detections).

In this work, we developed a multi-scale, integrated species distribution model (hereafter, MS-iSDM) of relative abundance and occupancy for a species of North American bat (tricolored bat) based on an inhomogeneous spatial point pattern process (Fig. 1). We used a change-of-support formulation via a shared hierarchical spatial mesh (the NABat master sample) and joint likelihood approaches to construct and link complex observation models (with false-negatives, false-positives, availability bias) of each data set (Fig. 1). Next, we used a seasonal migratory connectivity covariate to link population dynamics and impacts across seasons (Fig. 1), for which we expected a strong positive relationship with summer populations. We applied this model to 11 years of data for the tricolored bat to improve population inferences and provide a comprehensive inference on status and trends across population metrics (occupancy and relative abundance, winter and summer). Predictions from the MS-iSDM specifically represent the species distribution and trends over time in the ‘pre-volancy’ period (i.e., May 1st - July 15^th^, before newborn of the year can fly), which is the primary time period targeted for monitoring in the maternity season^8,18^. Following best practices in applying unmarked abundance models to monitoring data from complex ecological systems, we conservatively interpret abundance for tricolored bat as relative abundance (Refer to “Methods” for more details).

We examined population inferences for the tricolored bat under the MS-iSDM using all available data from 2012 to 2022, while describing how trends in occupancy and relative abundance compare in space and time with a suite of multi-scale environmental predictors (abiotic and biotic), with observed population declines due to the advance of WNS, and with each other. Our study provides a rare, empirical comparison using a shared data set and inferential framework of spatiotemporal trends in occupancy and relative abundances across the range of a species undergoing severe population declines. While there are an increasing number of studies on species distributions from range-wide monitoring efforts^29,30^, species distribution dynamics in response to novel wildlife diseases at this scale are largely unexplored given the aforementioned challenges of monitoring. We expected that our integrated distribution model would reconcile the apparent asynchrony in trends observed to date in occupancy and abundance-based metrics for tricolored bat^25^. We also expected to find a positive but saturating abundance-occupancy relationship^3^ in annual estimates aggregated at the range-level, given that such relationships are more commonly observed in species which are widespread, species with metapopulation dynamics, or species undergoing population declines^3^.

Next, we formally investigated the value of using combined inference under a single inferential paradigm relative to multiple independent analyses by fitting data from 2016 to 2022 for each data set independently and comparing inferences to those under the fully integrated model. We compared point estimates and uncertainties of environmental relationships, population trends, and mapped occupancy and relative abundance distributions. We expected that our integrated model would reduce uncertainty in estimates and predicted species distributions. Our model can be used for data integration of other bat species in North America and is also applicable to other macro scale ecological monitoring programs which collect multiple sources of data. In particular, the ability of our model to simultaneously account for false-negatives, false-positives, differential sampling exposure, occupancy-abundance relationships, and seasonal population dynamics represents important advancements in species distribution modeling.

## Results

### Sampling effort by field method type

Our modeling effort included data from 120,014 observation nights from 4 different field methods (mobile transect acoustics, stationary acoustics, MLE-acoustics [i.e., stationary acoustics which used a ‘maximum likelihood estimator’ approach to remove suspected false-positives], and live-captures). Total effort (observation nights) by field method type is reported in Table 1. Total effort (observation nights and grid cells) by field method type and year are reported in Supplementary Table 1, and sampled locations are mapped by field method type and year in Supplementary Fig. 1.

**Table 1:** Number of sample nights per monitoring data type, and assumptions on observation biases (imperfect detection, false-positives) for each (stationary acoustics, mobile acoustics, USFWS MLE acoustics, and live-capture)

Data type	Number of sample nights by location	Prone to imperfect detection	Prone to false-positives (misclassification)
stationary auto ID only	82,157	yes	yes
stationary auto ID and manual review	3753	yes	no
mobile Auto ID only	20,343	yes	yes
mobile auto ID and manual review	1147	yes	no
USFWS stationary acoustic mle	611	yes	no
Capture (5 km)	12,003	yes	no

Auto ID represents acoustic records classified to species using automated classification software. Manual review entails a subset of auto IDs which are reviewed (number depicted) of which some amount are also confirmed.

### Inferences from the MS-iSDM (2012–2022)

**Fig. 2:**
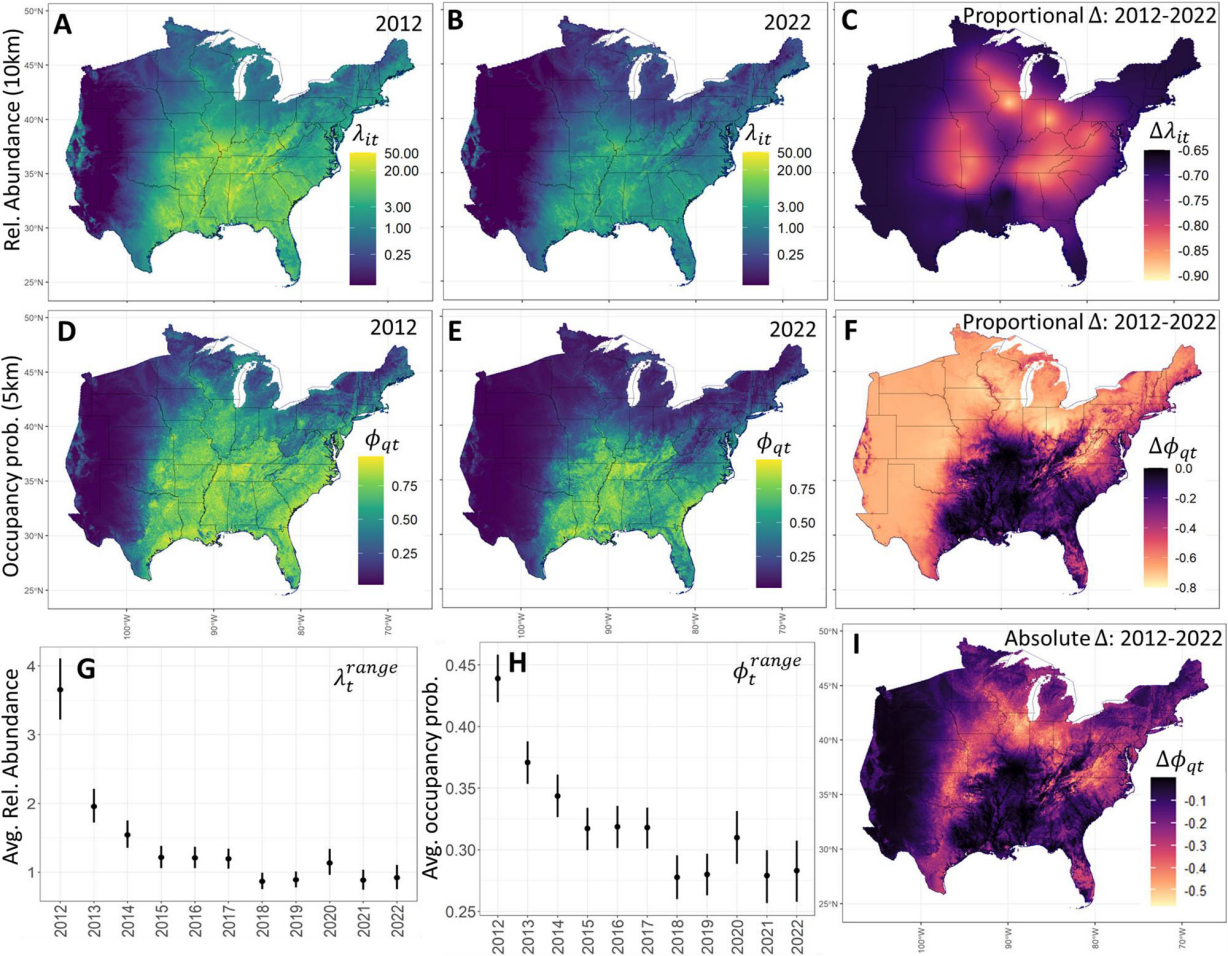
Range-wide population status and trend inferences from the multi-scale integrated species distribution model (MS-iSDM) for the tricolored bat from 2012 to 2022 in the pre-volancy season (May 1–July 15). Let *i* indicate NABat grid cells (10km x 10-km), *q* indicate quadrants (5 km x 5 km), *t* indicate the pre-volancy season each year. Inferences include the predicted species distribution of relative abundance (${\lambda }_{{it}}$, **A**: 2012, **B**: 2022, 10-km x 10-km grid cells), proportional rate of change in relative abundance between 2012 and 2022 (**C**), occupancy probabilities (${\phi }_{{qt}}$, **D**: 2012, **E**: 2022, 5-km x 5-km quadrants), proportional rate of change in occupancy probabilities between 2012 and 2022 (**F**), range-wide estimates of the population time series (**G**: ${\lambda }_{t}^{{range}}$, average relative abundance; **H**: ${\phi }_{t}^{{range}}$, average occupancy probability), and the absolute differences in occupancy probabilities ${\phi }_{{qt}}$ between 2012 and 2022 (**I**). The total proportional rate of change in range-wide average relative abundance (${\lambda }_{t}^{{range}}$, 10-km x 10-km) was estimated at –74.8% (–79.7% to –69.3%), while the total change in range-wide average occupancy (${\phi }_{y}^{{range}}$, 5-km x 5-km) was estimated at –35.5% (–41.1% to –30.2%) with probabilities >0.999 that trends were negative. Points depict posterior means, and error bars depict 95% Bayesian credible intervals. State polygons are from GADM (Global Administrative Areas, 2018)^68^.

Results and predictions from the MS-iSDM provide range-wide population inferences for tricolored bat from 2012 to 2022 in the pre-volancy season (May 1—July 15) at multiple scales (Fig. 2). The predicted distribution of relative abundance (${\lambda }_{{it}}$, 10-km x 10-km grids) for tricolored bat in 2012 (Fig. 2A) and 2022 (Fig. 2B) reveals population declines corresponding in space (grid *i*) and time (*t*) with winter WNS impacts. Proportional rates of total change between 2012–2022 in grid cells ranged between –0.65 and –0.90 (Fig. 2C). The predicted distribution of occupancy probability (${\phi }_{{qt}}$, 5-km x 5-km quads) in 2012 (Fig. 2D) and 2022 (Fig. 2E) also reveal population declines in tricolored bat. Proportional rates of total change in occupancy probabilities (Fig. 2F) ranged between 0.00 and -0.80, with the largest declines estimated along the periphery of the range (where relative abundances were low in 2012). Absolute declines in occupancy probabilities (Fig. 2I) were largest along the periphery of the interior (i.e., where relative abundances were at intermediate levels in 2012).

**Fig. 3:**
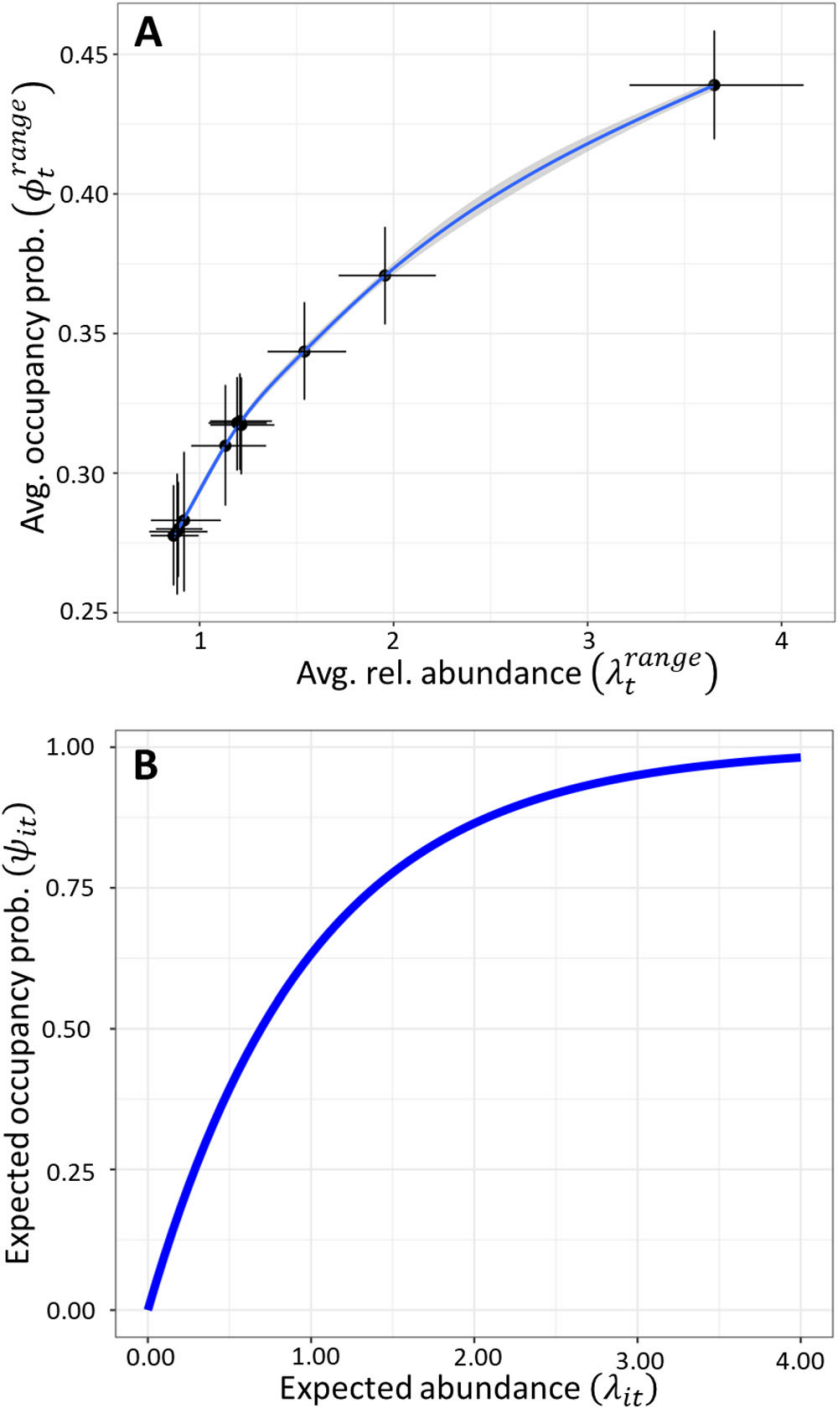
Estimated and deterministic relationships between relative abundance and occupancy probability (abundance-occupancy relationships) for tricolored bat from 2012 to 2022 at range-wide and local scales. **A** Estimated relationship between average range-wide relative abundance and average range-wide occupancy probability for the tricolored bat each year. Points depict posterior means, and error bars depict 95% Bayesian credible intervals. The best-fit-curve of point estimates from a generalized additive model is depicted as the blue line (mean) and gray shading (95% confidence interval). **B** Deterministic relationship between expected abundance and occupancy probability at the grid cell level based on the shared probability of zero.

Range-wide estimates of the average relative abundance from 2012–2022 (Fig. 2G: ${\lambda }_{t}^{{range}}$, averaged over all 10-km x 10-km grid cells each year) indicate overall population declines, with a proportional rate of total change estimated at –74.8% (–79.7% to –69.3%). Range-wide estimates of the average occupancy probability from 2012 to 2022 (Fig. 2H: ${\phi }_{t}^{{range}}$, averaged over all 5-km x 5-km quads each year) also indicate overall declines, with proportional rates of total change estimated at –35.5% (–41.1% to –30.2%). Similarly, the proportional rate of total change in average grid cell occupancy (averaged across all 10-km x 10-km grid cells) was estimated as –36.6% (–42.3% to 31.2%). Though each trend metric showed strong evidence of population decline (probability >0.999), the decline in range-wide relative abundance was approximately 2.1 times greater than the decline in range-wide occupancy probability. The abundance-occupancy relationship^3^ (Fig. 3A) between range-wide average relative abundance (Fig. 2G: ${\lambda }_{t}^{{range}}$) and range-wide average occupancy (Fig. 2H: ${\phi }_{t}^{{range}}$, 5 km quads) over time was positive, non-linear, and saturating (Fig. 3A). At the individual grid cell level (10-km x 10-km), the relationship between expected abundance $\left({\lambda }_{{it}}\right)$ and the occupancy probability is deterministic (Fig. 3B) based on probability of zero under a Poisson distribution.

**Fig. 4:**
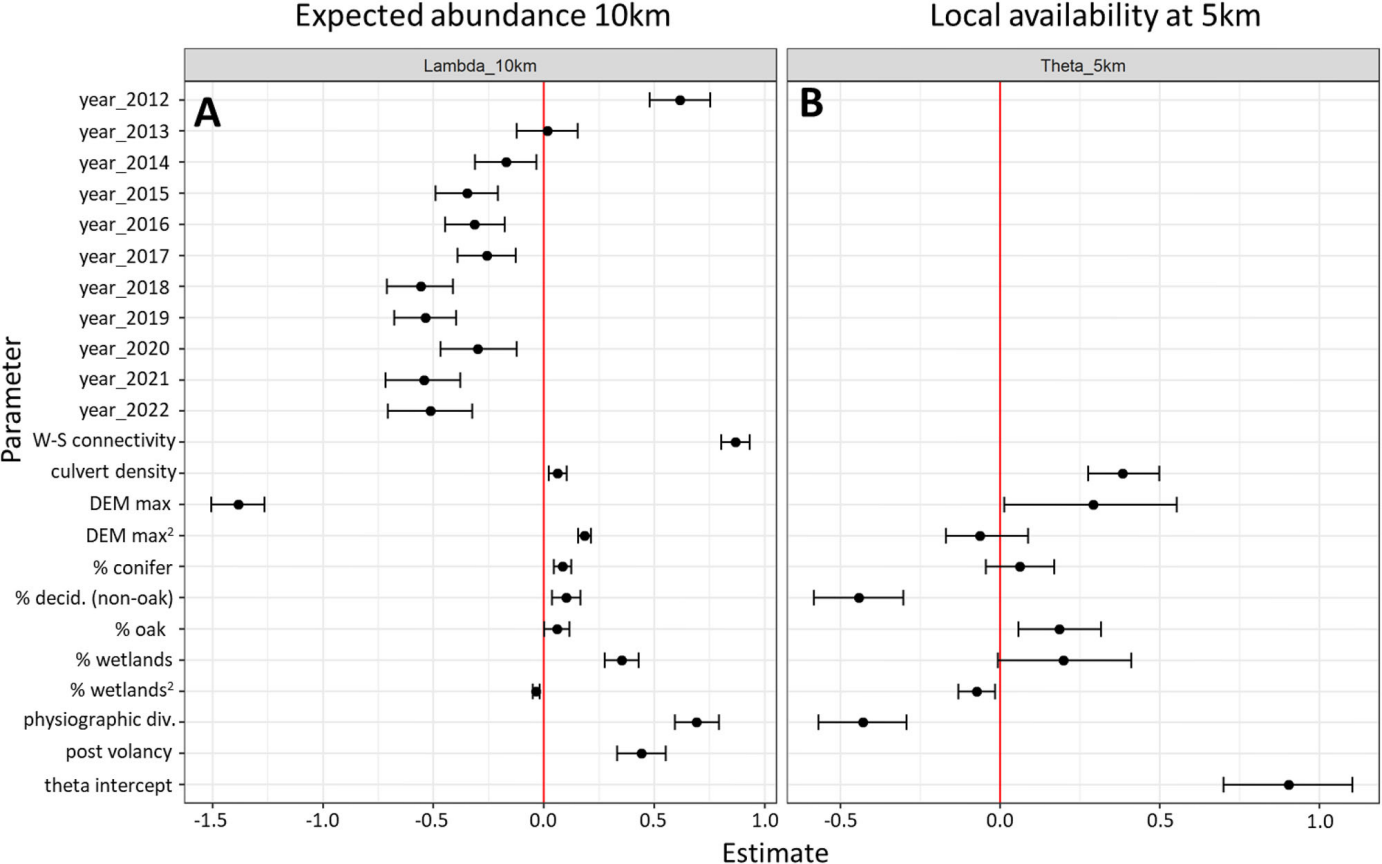
Covariate effect estimates for tricolored bat under the multi-scale integrated species distribution model (MS-iSDM) using data from 2012 to 2022 for expected abundance at the 10-km x 10-km scale, and in local availability at the 5-km x 5-km scale. **A** Parameter estimates for expected abundance at the 10-km x 10-km scale. **B** Parameter estimates for local availability at the 5-km x 5-km quadrant scale, conditional on occupancy at the 10-km x 10-km scale. Points depict posterior means, and error bars depict 95% credible intervals. DEM represents maximum elevation and 'W-S connectivity' represents winter-to-summer migratory connectivity.

Estimated ecological covariate effects under the MS-iSDM (Fig. 4) reveal how multi-scale ecological relationships combine to predict the species distribution and trends over time (Fig. 2). For example, we found positive effects of migratory connectivity (Supplementary Fig. 2), culvert density, physiographic diversity, all three forest cover types (conifer, deciduous-oak, deciduous-non-oak), and the post-volancy effect (change between pre-volancy and post-volancy seasons) on relative abundance at the 10-km x 10-km scale (Fig. 4A). Wetlands cover had a positive linear term and negative quadratic term, while maximum elevation (DEM) a negative linear term and positive quadratic term. For local availability at the 5-km x 5-km scale (Fig. 4B), we found positive effects of culvert density, oak cover, conifer cover, maximum elevation, and wetlands cover (linear and quadratic); and negative effects for physiographic diversity and deciduous (non-oak) cover. Additional results from the full MS-iSDM analysis, including estimates for detection covariates (Supplementary Figs. 3–5) and estimates of ${\lambda }_{{it}},{\theta }_{{qt}},$ and ${\phi }_{{qt}}$ for sampled locations (Supplementary Fig. 6) are provided in the supporting materials (Appendix S3: Supplementary Results). A full set of results including model estimates, abundance and occupancy predictions, and trends over time are also provided as a US Geological Survey data release^31^.

Model validation for the full iSDM (2012–2022) evaluated on predictive occupancy resulted in an average Area Under the Curve (AUC) of 0.801 for presence/absence at the 5 km scale and 0.883 at the 10-km x 10-km scale (in-sample). A visual assessment of posterior predictive checks for the expected vs observed counts (in-sample) of mobile acoustics and stationary acoustics data is reported in Appendix S3 (Supplementary Fig. 8). The average occupancy probability of a leave-out data set (*n* = 206) of known presences was 0.86 at the 10 km scale, and 0.70 at the 5-km x 5-km scale. Comparing the distributions of predicted occupancy probabilities for the leave-out presence data to the distribution across all sampled quads and years (Supplementary Fig. 9) demonstrated a consistently higher predicted occupancy probabilities compared to the background rate.

### Integrated vs multiple independent model comparisons

**Fig. 5:**
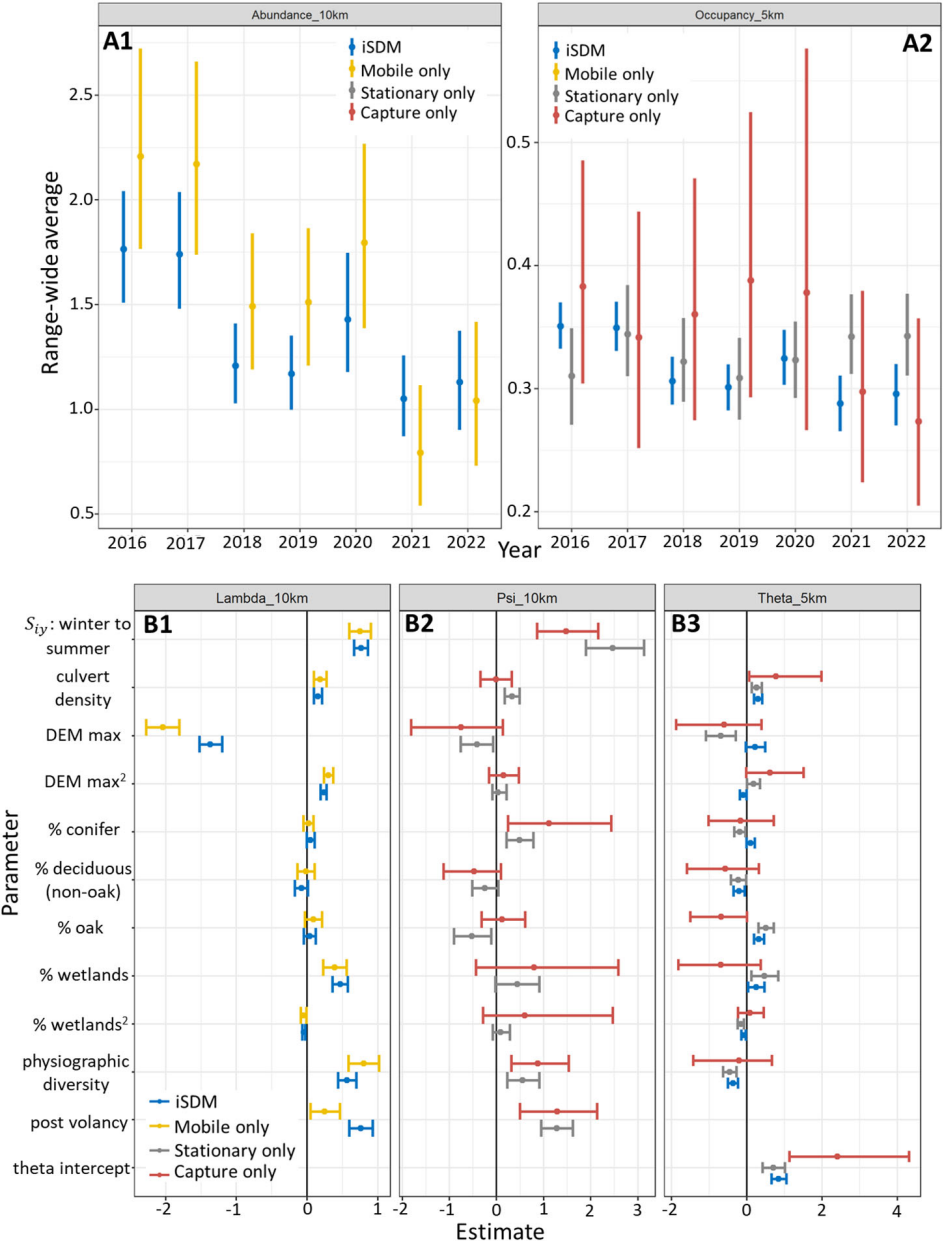
Combined analysis under the multi-scale integrated species distribution model (MS-iSDM) improves population inferences for tricolored bat relative to multiple, independent lines of evidence. **A1**: Estimates of range level population status in relative abundance (average relative abundance) using the MS-iSDM compared with only using mobile transects. **A2**: Estimates of range level population status in species occupancy probability (average occupancy probability) under the MS-iSDM compared with only using stationary acoustics or only live-capture data. In addition to reduced uncertainty in population status and trends estimates, the data integration acts as a smother of observed trends to produce consensus-estimates over time in both occupancy and abundance. **B**: Covariate effect estimates under each model (MS-iSDM and independent) for expected abundance at the 10-km x 10-km scale (**B1**: MS-iSDM versus independent mobile transects), occupancy probability at the 10-km x 10-km (**B2**: independent stationary and capture models), and in local availability at the 5-km x 5-km scale given conditional on occupancy at the 10-km x 10-km scale (**B3**: MS-iSDM versus independent stationary and capture models). Points depict posterior means, and error bars depict 95% credible intervals. DEM represents maximum elevation and ${S}_{{iy}}$ represents seasonal (winter to summer) migratory connectivity.

**Fig. 6:**
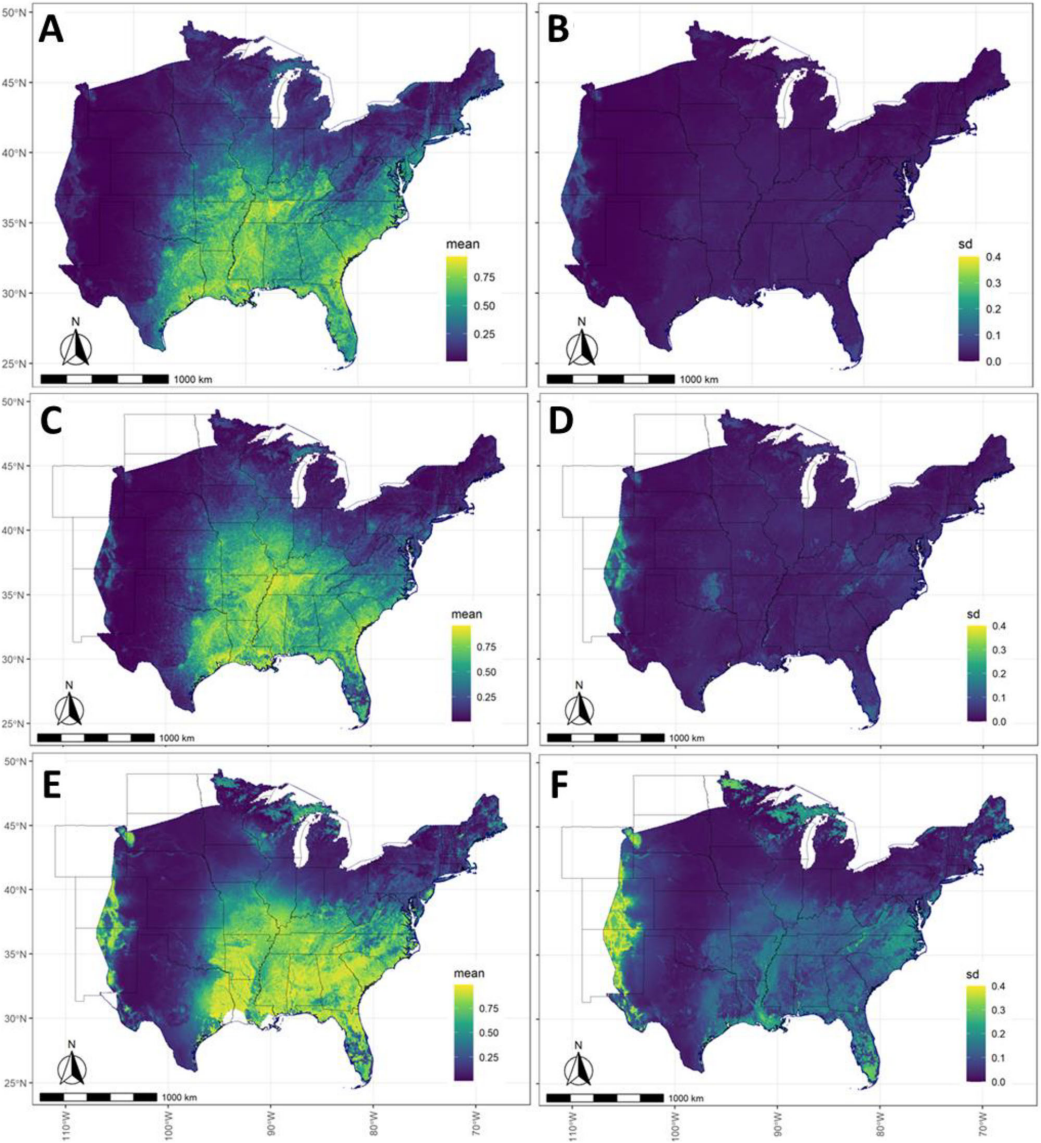
Model predictions(occupancy probabilities and uncertainties) for tricolored bat in 2022 at the 5-km x 5-km scale under the multi-scale integrated species distribution model (MS-iSDM) compared to multi-scale occupancy models fit using only stationary acoustics or live-capture data. Predictions from the MS-iSDM (**A**: mean occupancy probabilities, **B**: standard deviations) were less uncertain than those from the models using only stationary acoustics (**C**: mean occupancy probabilities, **D**: standard deviations) or only using live-capture data (**E**: mean occupancy probabilities, **F**: standard deviations). State polygons are from GADM (Global Administrative Areas, 2018)^68^.

A comparison of inferences obtained under the fully integrated model and each individual model fit independently (one for each field method) using data from 2016 – 2022 demonstrated the value of combined inference (Figs. 5, 6). These benefits included both reduced uncertainties and weighted-averaging of the comprehensive estimates compared with those under each independent model, including range-wide population status estimates and their trajectories over time (Fig. 5-A1: ${\lambda }_{t}^{{range}}$, average relative abundance across all 10-km x 10-km grid cells; Fig. 5-A2: ${\phi }_{t}^{{range}}$, average occupancy probabilities across all 5-km x 5-km quadrants), and multi-scale covariate effects for ecological predictors (Fig. 5B). Uncertainty measures (95% Bayesian credible interval widths) in range-wide average abundance estimates were reduced by 31–46% with the integrated model compared to mobile acoustics alone. The integrated model also reduced uncertainty measures in the average range-wide occupancy estimates by 67–86% compared to the capture-only model and 25–52% compared to the stationary-acoustics-only model. Because models without mobile transects estimated occupancy probabilities ($\psi$) instead of expected abundance ($\lambda$) at the 10-km x 10-km scale, covariate estimates are provided for either $\lambda$ or $\psi$ at the 10-km x 10-km scale depending on the underlying model (Fig. 5).

## Discussion

In this work, we developed a multi-scale, false-positive, integrated species distribution model (MS-iSDM) for North American bats which combines monitoring information across survey method types, spatial scales, ecological states, years, and seasons to improve population inferences on species occupancy and abundance across its range. We achieved this by building upon recent statistical (e.g., joint likelihood methods for data integration) and ecological (e.g., migratory connectivity to link seasonal distributions) advances for modeling distributions of cryptic species with seasonal and migratory life cycles. We applied this model to 11 years of data for the tricolored bat to gain a better understanding of population status and trends in abundance and occupancy, relationships with ecological predictors, and the abundance-occupancy relationship over time. Inferences under this model demonstrated clear population declines between 2012 and 2022 as captured by trends in relative abundance and occupancy, which varied spatially and temporally based on multidimensional nature of the modeled niche (covariate effects), spatial variation in population dynamics, and a non-linear abundance-occupancy relationship at local scales (Fig. 3B). By comparing inferences of the fully integrated model to independent analyses of each data set using a confined temporal scope (2016–2022), we demonstrated improved population inferences for the tricolored bat using combined inference relative to using multiple lines of evidence analyzed independently.

We found strong evidence (probabilities >0.999 that trends were negative) of population declines in the tricolored bat from 2012 to 2022 in both range-wide relative abundance (total change: mean = –74.8%) and occupancy (total change: mean = –35.5%). This reflects previous findings in bat distribution and trend modeling that occupancy is a less sensitive indicator of population change than relative abundance^18,19^, or activity^25^. It also highlights that even catastrophic declines in abundance may result in only modest declines in occupancy probability for wide-spread and abundant species. At the grid cell level, we found the strongest declines in relative abundance (approximately -90%) corresponded spatially with the most severe declines in winter abundance along the advancing wave of WNS impacts^14^ in the interior of the range (Fig. 2). However, occupancy probabilities were largely stable in these regions because relative abundances were initially high, and occupancy probabilities remained near one (Fig. 2). Rather, the strongest proportional declines in occupancy probability were observed along the periphery of the species range where starting abundances were already low. Absolute change in occupancy probability (Fig. 2I) declined most along the periphery of areas with high relative abundance, in and near locations with winter WNS impacts and moderately high (e.g., 0.25–0.75) occupancy probabilities in 2012.

While macro-scale trends in summer occupancy^17^ and relative abundance^18^ have been previously modeled for tricolored bat, this is the first time both state variables were analyzed using an integrated framework. Encouragingly, overall trend estimates for occupancy and relative abundance from this work are similar in direction and magnitude as those found in previous work^17,18^ despite some differences in monitoring data, spatial and temporal scope, predictive covariates, and statistical methods. Furthermore, the finding of differing spatiotemporal trends in occupancy and population impacts agrees with prior work on tricolored bats trends^17,18,25^. However, providing estimates of both occupancy and relative abundance from a single inferential framework (i.e., integrated distribution model) leads to additional insights into population ecology.

For example, the asynchrony between occupancy trends and population impacts is explained as an emergent pattern of spatiotemporal variation in abundance in the integrated distribution model. It also explains an apparent conundrum for conservation management, i.e., how tricolored bats can remain widespread in occupancy (use by at least one bat) despite severe population impacts. Put simply, local occupancy probability declines non-linearly with abundance (Fig. 3B) and will be mostly stable in locations with high initial abundances despite large population declines. Occupancy trends will only become apparent locally when abundances approach zero (Fig. 3B). Spatiotemporal variation in abundance interacts with the saturating relationship between occupancy probability and abundance, and in this case, also metapopulation connectivity to predict the observed range dynamics in tricolored bat occupancy (Fig. 2). Indeed, such a pattern is predicted from metapopulation theory when Allee effects^32^ are present. Populations in the interior of the range could be buffered from local extinction due to rescue effects while populations along the range periphery decline due to smaller initial population sizes and reduced dispersal flux from the interior^3,32^.

As expected, we found a positive, non-linear, and saturating relationship between range-wide relative abundance and occupancy over time (intraspecific abundance-occupancy relationship, Fig. 3A). Our integrated distribution model includes a combination of all four categories of mechanisms (statistical, resource, range-position, and population dynamics) which have been suggested to produce positive intraspecific abundance-occupancy relationships^3^. The latent spatial point pattern process reflects “statistical mechanisms” (i.e., shared probability of zero for abundance and occupancy given an assumed spatial mesh and statistical distribution) while predictive covariates (abiotic and biotic) reflect resource mechanisms. The inclusion of a metapopulation based “seasonal migratory connectivity” covariate reflected both “population dynamic” and “range-position” mechanisms.

Multi-scale ecological relationships on abundance and local occurrence (Figs. 4,  5) combined to predict the species distribution (Figs. 2,  6) and were generally in line with our expectations (Supplementary Table 2). When interpreting covariate effects (within and across scales), it is important to remember they are estimated conditional on all other covariate effects in the model. We found a positive effect for culvert density on the relative abundance and occupancy of tricolored bat, which may reflect their use as potential roosting habitat in the winter and summer. Based on long-held hypotheses concerning landscape complexity and bat distributions^33^, and findings from previous work^17,18^, we expected positive effects of physiographic diversity for both ecological states and spatial scales. While we found a positive effect on relative abundance at the 10-km x 10-km scale, we also found negative effects on availability at the 5-km x 5-km scale. This could possibly reflect multi-scale habitat selection processes for summer roosts, foraging grounds, and home ranges, where physiographic diversity may support higher abundances at larger spatial scales (e.g., due to complementary resources across trophic levels), while areas with the highest landscape complexity might be avoided at local scales.

We found that winter-to-summer migratory connectivity had the strongest positive effect of all predictors on abundance (Figs. 4, 5), demonstrating the utility of this linkage between winter and summer populations. This covariate importantly reflects a source of ‘non-stationarity’ in the population demography across a spatiotemporal gradient^34^ driven by population impacts from a rapidly expanding disease, which would have likely biased population inferences if not accounted for. Seasonal population dynamics are becoming increasingly recognized as important for modeling species distributions, especially for migratory species^12,13,34^. Full annual cycle models have been discussed in the literature^35,36^, but are especially challenging for species with large ranges. One important consideration is that we assumed no directionality in migration (a common assumption of regionally migratory bats^37^ and metapopulation connectivity^26,27^), although isotypic evidence for tricolored bats^38,39^ suggests that migration may be variable in both directionality and distance traveled. Our framework could be used in future studies to quantify and evaluate support for different migratory hypotheses such as differences in migration distances or directionality throughout the species range, though model selection in integrated models remains a challenging topic^12^.

Additional improvements in model accuracy are expected by combining complementary data sets which do not suffer from the same observational biases^12,13,40^. By integrating across multiple data types, we endeavored to overcome the individual limitations of each data set by modeling a single underlying truth which is shared between observation models. We demonstrated that combined inference under the integrated model (as opposed to multiple lines of evidence) reduced uncertainty and provided a comprehensive, appropriately weighted ‘consensus’ estimates^11^ of population trends over time (Fig. 5A), covariate effects (Fig. 5B), and spatial patterns (Fig. 6). This ‘smoothing’ of estimates was observed in both relative abundance and occupancy estimates (Figs. 5,  6), highlighting the flow of information across ecological states, spatial scales, and monitoring data sets.

Data integration also facilitated an increased spatiotemporal scope of inference. Slightly stronger estimated effects of winter-to-summer connectivity under the integrated model (Fig. 5-B1) could reflect increased (and more representative) spatial coverage relative to mobile transects alone. A stronger post-volancy effect (i.e., apparent birth rate) likely reflects a greater number of sampling events in the post-volancy season (i.e., when juvenile bats begin to fly and become detectable by acoustics) when data streams are integrated. Furthermore, estimating the relationships between data types and ecological states in years where each was common (e.g., 2016 on) allowed us to extend estimates under the full model to years when only some monitoring types were common. We included data collected before 2012–2015 to better capture WNS impacts which occurred before the NABat Monitoring Protocol^8^ was officially established. Had we only analyzed the data through 2016 based on the most limited data set, we would have missed severe population declines which occurred between 2012 and 2015. These data, and those collected opportunistically (e.g., capture data), were less ‘structured’ and less representative than those collected under the NABat protocol. Including such data could potentially result in some amount of bias in earlier years due to non-representative sampling^41^, especially if data sets are analyzed independently.

This work represents the most comprehensive population and species distribution analysis to date for the imperiled tricolored bat in the maternity season, integrating information across both winter and summer. Results from this work have been used to inform U.S. Fish and Wildlife Service’s (USFWS) environmental review process based on where the species is likely to occur^42^. Furthermore, predicted maps of relative abundance and occupancy probability^42^ (Fig. 2, Supplementary Fig. 7) can be used in environmental reviews to determine the proportion of the species abundance or distribution that might be at risk of some proposed action in a region of interest. Finally, maps of occupancy probability, relative abundance, and trends over time can be integrated with formal or informal species status assessments or spatial conservation planning efforts.

Standard presence-only species distribution models do not typically account for observation bias (false-negatives or false-positives), though data integration has increasingly been used to account for imperfect detection^12,13,24,40,43,44^. One notable exception accounted for both false-negatives and false-positives using a non-identifiable mixture^45^ which required at least two additional data sources (a subset of reviewed data and a second data stream without false-positives) or parameter constraints to make the model identifiable. Our false-positive N-mixture and occupancy models—formulated as count-detection models with a hypergeometric review process—overcome this limitation^18,20^ and do not require a second source of unambiguous detections. Despite this, our inclusion of additional data sources without false-positives (live-capture, MLE-acoustics) likely further improved model accuracy. Furthermore, our multi-scale iSDM formulation helped to better account for differential sampling exposure^46^ (availability) between data sets compared to the typical single scale models used for iSDMs^12,13,24,40,43,44^.

Our work had several limitations including: (1) less representative sampling in monitoring data sets before 2016, (2) long computational run times which limited the number of alternative model comparisons, and (3) the use of broad and simplifying assumptions when constructing the winter-to-summer migratory connectivity metric resulting from a limited understanding of tricolored bat migration throughout its range. One potential source of improvement would be using local habitat covariates in proximity of transects to inform any heterogeneity of availability within a grid cell. Accounting for spatial autocorrelation, especially in the relative abundance trends over time, may help account for additional non-stationarity if computational limitations can be overcome. Future extensions which include presence only summer roost locations or counts could provide additional information on summer abundance. Finally, extending this model to include explicit seasonal population dynamics (e.g., apparent birth and survival processes^47^) may provide additional insights into the demography of bats if methodological challenges can be overcome.

## Conclusion

In this work, we developed a first of its kind model (multi-scale, false-positive, iSDM) for North American bats which integrates monitoring information across survey types, spatial scales, ecological states, years, and seasons to improve population inferences on species occupancy and abundance across a species range. We demonstrated how multiple data integration techniques can be combined to model cross-seasonal species distributions and population trends informed by migratory connectivity from data sets with complex observational processes (false-negatives, false-positives, availability bias). Ultimately, our work takes an important step forward in applying data integration approaches to modeling macro scale distributions of species with cryptic, seasonal, and migratory life histories; and in modeling the full annual cycle of bats in North America.

## Methods

### Probabilistic sampling grid and protocol

The NABat master sample^8^ is based on a grid-based sample frame with 10-km × 10-km cells (NABat grid cell), with each grid further sub-divided into 5-km × 5-km quadrants (quads, Fig. 1). The grid-based sample frame serves as a multi-scale, hierarchical ‘mesh’ (quads within grid cells) to facilitate the change-of-support^48^ of across various spatial scales. To achieve NABat objectives, a probabilistic survey design for site selection was established to support defensible range-wide inferences, encourage collaboration, and facilitate data integration among multiple states, regions, and agencies^8^. While priority sampling order is stressed for selecting grid cells for monitoring, unique considerations and constraints of each monitoring partner weigh into the final selections^10^.

### Field method types

We analyzed data from the NABat database^49–51^ which were collected between 2012–2022. Monitoring protocols for NABat built upon previous efforts established by program partners, and are documented in guidance documents^8,52,53^. Field monitoring methods consist of acoustic monitoring in the summer (point sampling and transects), live-captures in the summer, and counts of winter colony sizes typically obtained at hibernacula (Fig. 1). Autonomous recording units (ARUs) were deployed at fixed locations (stationary acoustic surveys) or affixed to a vehicle that traveled along a road transect (mobile acoustic surveys). Temporal and spatial replication of surveys, along with manual vetting for subsets of records, provides information to account for both false-negatives and false-positives^8,18,20^. The total count of auto IDs (species detections by automated classification software), and the total number of auto IDs which were manually reviewed and confirmed as the species of interest, were treated as the response variables for each survey location, night, and method (Supplementary Methods 1).

Live-capture data contributed to NABat are reported as the total number of bats of each species captured for each location and night. Non-detections were inferred across all capture survey efforts in the database whenever sampling effort was reported (at least one other species was caught) and no tricolored bats were captured. Winter season data are comprised of colony counts at winter roosts (often with temporal replication), such as caves, mines, culverts, bridges, or other human structures^14^. A separate status and trends analysis using Bayesian hierarchical modeling has provided modeled estimates of abundance each year in hibernaculum^19^. Data from summer maternity roosts were not included in this analysis due to data sparsity.

### Multi-scale integrated species distribution model (MS-iSDM)

Following prior work^11^, we constructed a joint likelihood approach to accommodate multiple data sources and observation processes based on a common spatial point process for latent abundance (Fig. 1B). The NABat grid-based sample frame serves as a multi-scale, hierarchical ‘mesh’ (5-km × 5-km quads within 10-km x 10-km grid cells) that defines the spatial resolutions at which we define occupancy and abundance. We assume an inhomogeneous Poisson point pattern process^24^ for latent abundance, estimated for each 10-km x 10-km grid cell *i* and for each time period *t* as: ${N}_{{it}} \sim {{{\rm{Poisson}}}}\left({\lambda }_{{it}}\right)$. Because the summer maternity season for bats is bisected by the birth pulse and volancy of newborns, we subdivide year (corresponding with the summertime season each year) into two sub-periods: pre-volancy and post-volancy, using July 15^th^ as the cutoff^8,18^. Thus, *t* corresponds with time period (year and sub-period, arranged in chronological order). We model the expected abundance, ${\lambda }_{{it}}$, as a function of spatial, temporal, and spatiotemporal covariates using a logit link:

$$\log \left({\lambda }_{{it}}\right)={{{{\boldsymbol{x}}}}}_{{{{\boldsymbol{i}}}}}^{\prime}{{{\boldsymbol{\beta }}}}+{b}_{y}+{\beta }_{{post}}\times {pos}{t}_{{it}}+c \times {S}_{{iy}}$$

where ${{{{\boldsymbol{x}}}}}_{{{{\boldsymbol{i}}}}}^{{{{\prime}}}}$ is a row vector of grid cell-level covariate values, ${{{\boldsymbol{\beta }}}}$ is a vector of covariate coefficients, ${b}_{y}$ is the temporal random intercept for population abundance in the pre-volancy season (adult population) each year. To share information between years in abundance, we assume an AR^1^ process on the intercept over time, $[{b}_{y}]={AR}(1)$, for years y = 1, · · · *Y*, with Y denoting the number of years available. Furthermore, ${pos}{t}_{{it}}$ is a binary indicator for the post-volancy season (sub-period = 2 each year), ${\beta }_{{post}}$ represents the post-volancy effect on abundance, or the apparent birth rate (i.e., the average rate of increase in abundance after the birth pulse) each year. The variable $c$ is the migratory connectivity coefficient, and ${S}_{{iy}}$ is a measure of potential migratory connectivity quantified as abundance weighted dispersal flux (Fig. 1; See *Integrating across seasonal distributions*).

As proposed in prior work^11^, we link presence/absence datasets to the latent abundance process via the deterministic relationship between the occupancy state ${z}_{{it}}$ and abundance ${N}_{{it}}$: ${z}_{{it}}=I\left({N}_{{it}} > 0\right)$ (Fig. 1). The derived occupancy probability (presence of at least one individual) given the latent abundance intensity ${\lambda }_{{it}}$ is $1-\exp \left(-{\lambda }_{{it}}\right),$ and the probability of absence (no individuals present) is $\exp \left(-{\lambda }_{{it}}\right)$. Because bats are mobile species, abundance ${N}_{{it}}$ represents the total number of animals which ‘use’ (for any reason) any portion of a grid cell, while the occupancy probability is the probability of ‘use’ by at least one individual, over each time period *t* (Fig. 1- B.1 and Fig. 1-B.2).

### Multi-scale abundance

We use a multi-scale abundance approach (Fig. 1-B.1 and Fig. 1-B.3) which links the abundance of bats in a 10-km x 10-km grid cell ${N}_{{it}}$ with the abundance of bats ${M}_{{kt}}$ exposed to sampling along each transect (i.e., those with home ranges intersecting the transect) based on sampling exposure rate ${\theta }_{k}^{{transect}}$ (the proportion of bats in a grid cell exposed to sampling). Like prior work^18,54^, we model the ${\theta }_{k}^{{transect}}$ as an exponential function of the inverse transect length: $\log \left({\theta }_{k}^{{transect}}\right)=-{\beta }_{{tl}}\times \frac{1}{{L}_{k}}$, where ${\beta }_{{tl}} > 0$. This results in a saturating function, with the rate of saturation determined by the slope of ${\beta }_{{tl}}$. We model ${M}_{{kt}}$ conditionally as a binomial sample of grid cell level abundance ${{{{\rm{N}}}}}_{{{{\rm{it}}}}}$ given the sampling exposure rate: ${M}_{{kt}} \sim {{{\rm{Bin}}}}\left({{{{\rm{N}}}}}_{{{{\rm{it}}}}},{\theta }_{k}^{{transect}}\right)$. This facilitated conditional inferences for *N*_*it*_ (in addition to *M*_*kt*_) based on data from each individual monitoring type (e.g., mobile transacts), while accounting for co-located observations within and between data types (e.g., site-level confirmation).

### Multi-scale occupancy

We used a multi-scale parameterization to represent the finer resolution of the nested 5-km x 5-km quads (denoted q) within a grid cell *i*, where ${q}_{{qt}} \sim {{{\rm{Bernoulli}}}}({{{{\rm{\theta }}}}}_{{{{\rm{qt}}}}}\times {z}_{{it}})$ and ${{{\rm{logit}}}}({\theta }_{{qt}})={w}^{{\prime} }\alpha$ can include covariates at the resolution of a quad *q* that explains local availability or occurrence (Fig. 1-B.2 and Fig. 1-B.4). Here, ${\theta }_{{qt}}$ is the probability of local availability at the quad level given occupancy at the grid-cell level, and ${q}_{{qt}}$ is the occupancy state at the quad level. The unconditional probability of occupancy at the 5-km x 5-km scale $({\phi }_{{qt}})$ is defined as ${{{{\rm{\theta }}}}}_{{{{\rm{qt}}}}}\times \left(1-\exp \left(-{\lambda }_{{it}}\right)\right).$

### Spatial covariates on $\lambda$*and*$\theta$

We model spatial covariates on $\lambda$ and $\theta$ via their link functions to explain heterogeneity in occurrence and abundance at both the 10-km x 10-km and 5-km x 5-km scales. Covariates (Supplementary Table 2) were included based on evidence from previous NABat^17,18^, or because they were thought to be associated with summer roosting or foraging habitat, including land cover types (forest types, wetlands), abiotic factors (elevation, physiographic diversity), and culverts (which are potential roosting structures). Physiographic diversity is a measure of landscape complexity that considers multiple factors (multiscale topographic position, slope, aspect, parent material, continuous heat load). The proportion of forest cover by type (coniferous, oak, non-oak deciduous), the count of culverts, physiographic diversity, maximum elevation (with quadratic effects) and proportion of wetland cover (with quadratic effects) were included as predictors on both expected abundance (10-km) and local occurrence/availability (quad, 5-km x 5-km). Each covariate is further described in Supplementary Table 2, including its source, measured spatial extent, reasoning for inclusion, a priori expectations and preparation steps.

### Integrating across seasonal distributions

We include a winter-to-summer, migratory connectivity covariate measure (${S}_{{iy}}$, Fig. 1 panel A to panel B) on expected abundance ${\lambda }_{{it}}$, calculated as abundance weighted dispersal flux, to link winter and summer population distributions in space and time as in prior work^18^. The interpretation of this covariate is the expected number of seasonal migrants from winter populations to each summer grid cell each year. It was calculated as the sum of expected contributions of all winter colonies to each summer grid cell, based on the abundance estimate of each winter colony each year, and the probability of connectivity between each winter colony and grid cell (given an exponential migration kernel, the average migration distance, and the distance between each winter colony and grid cell, Supplementary Methods 6). An average seasonal migration distance of 101.1 km was used based on banding and telemetry data reported in the literature (summarized in Udell et al.^18^). For annual winter abundances, we used point estimates from a separate NABat analysis of winter counts^19^ which used a Bayesian hierarchical time series modeling approach to account for missing observations and observation error. Additional methods and figures of seasonal migratory connectivity (Supplementary Fig. 2) are included in Supplementary Methods 6. Following prior work^18^ we include the value for winter-to-summer connectivity $({S}_{{iy}})$ into the log-link for ${\lambda }_{{it}}$ after applying a ‘log plus 1’ and centering it (subtracting the mean over all sites and years) to improve convergence.

### Integrating across monitoring method types (Observation models)

Observations from each monitoring data type were integrated into the species distribution model at different relevant spatial scales (Fig. 1B) and different ecological states. We specified a different hierarchical observation model for each data type based on their inherent observation biases (e.g., detectability, sampling exposure, false-positives, Fig. 1C). We use a super-script to distinguish the different datatypes and their associated model-parameters: ${v}_{{ktj}}^{(m)}$ for mobile ARU observations; ${v}_{{qtj}}^{\left(s\right)}$ for stationary ARU observations; ${y}_{{qtj}}^{({cap})}$ for live capture; and ${y}_{{qtj}}^{\left({mle}\right)}$ for the small subset of stationary ARU observations which used the ‘MLE-method’ to remove suspected false-positives (Fig. 1).

### Mobile transect acoustic surveys

NABat mobile transect acoustics consist of temporally replicated surveys of acoustic data collected from ARUs mounted to vehicles, which are driven along transects (ideally 25–48 km in length and driven at a minimum speed of 32 km/hr^8^) within NABat grid cells. Mobile transect acoustics were primarily linked with abundance at the 10-km x 10-km scale based on a false-positive N-mixture model^18,20^. Given their speed, mobile transects were originally designed so that each bat detection could be reasonably assumed to correspond with a single individual, and support inferences via unmarked abundance models^8^. However, the assumption that animals are only detected once is no longer required for recent unmarked abundance methods which use a Poisson observation model (as opposed to a Binomial) for the count-detection process^18,55^.

We let ${v}_{{ktj}}^{(m)}$ denote the observed number of auto IDs classified as a tricolored bat recording during a nightly visit *j* to a transect $k$ during time period *t*. Following prior work^18^, we used a Poisson observation model for the count-detection process of auto IDs (${v}_{{ktj}}^{(m)}$, Fig. 1- C1) based on the transect level abundance ${M}_{{kt}}$ in each time period, the per capita detection rate ${\delta }_{{ktj}}$, and the false-positive rate ${\omega }_{{ktj}}$:

$${v}_{{ktj}}^{(m)} \sim {{{\rm{Poisson}}}}\left({\delta }_{{ktj}}^{\left(m\right)}\times {M}_{{kt}}+{\omega }_{{ktj}}^{\left(m\right)}\right)$$

The per-capita detection rate ${\delta }_{{ktj}}^{\left(m\right)}$ is the average number of true detections per individual in ${M}_{{kt}}$ each night *j*. The auto IDs are a combination true positives (denoted as ${{{{\rm{K}}}}}_{{ktj}}^{(m)}$, which are unknown and occur at a rate of ${\delta }_{{ktj}}^{m}\times {M}_{{kt}}$) and false positives (${{{{\rm{Q}}}}}_{{ktj}}^{(m)}$ that occur at a nightly rate of ${\omega }_{{ktj}}$). These latent parameters are estimated as: ${{{{\rm{K}}}}}_{{ktj}}^{(m)} \sim {{{\rm{Binomial}}}}\left({v}_{{ktj}}^{\left(m\right)},\frac{{\delta }_{{ktj}}^{\left(m\right)}\times {M}_{{kt}}}{{\delta }_{{ktj}}^{\left(m\right)}\times {M}_{{kt}}+{\omega }_{{ktj}}^{\left(m\right)}}\right)$, where $\frac{{\delta }_{{ktj}}^{\left(m\right)}\times {M}_{{kt}}}{{\delta }_{{ktj}}^{\left(m\right)}\times {M}_{{kt}}+{\omega }_{{ktj}}^{\left(m\right)}}$ is the true positive rate; and ${{{{\rm{Q}}}}}_{{ktj}}^{(m)}={{{{\rm{v}}}}}_{{ktj}}^{(m)}-{{{{\rm{K}}}}}_{{ktj}}^{(m)}$. A subset of manually reviewed ${n}_{{ktj}}^{\left(m\right)}$ and confirmed ${k}_{{ktj}}^{\left(m\right)}$ auto IDs were modeled via a hypergeometric formulation to provide the necessary information to estimate false-positive and true-positive rates^18,20^ (Supplementary Methods 2):

$${{{{\rm{k}}}}}_{{ktj}}^{(m)} \sim {{{\rm{Hypergeometric}}}}\left({n}_{{ktj}}^{(m)},{K}_{{ktj}}^{(m)},{{{{\rm{Q}}}}}_{{ktj}}^{(m)}\right)$$

Heterogeneity in observation parameters (${\delta }_{{ktj}}^{\left(m\right)}$ and ${\omega }_{{ktj}}^{\left(m\right)}$) was accounted for using a combination of spatiotemporal covariates and nested random effects (Fig. 1-C1, Supplementary Methods 2), which can help account for unexplained variation in space and time. We used three predictors of ${\delta }_{{ktj}}^{\left(m\right)}$ including: minimum air temperature, total precipitation, and day of year (with quadratic effects on day of year). Random slopes for each transect were also included for the linear and quadratic day of year effects (Supplementary Methods 2, as in Udell et al. ^18^). To account for differences among data contributors in the average false positive rates ${\omega }_{{ktj}}^{\left(m\right)}$, we included observation level (i.e., nightly) random effects nested within NABat project (i.e., data contributor)-level random effects (Supplementary Methods 2).

### Stationary acoustic surveys (with manual review)

NABat stationary acoustic surveys deploy 2–4 detectors within an NABat grid cell^8^. Ideally, one detector was placed in each 5-km x 5-km quad within a given NABat grid cell (Fig. 1-B4 and Fig. 1-C2). Detectors were deployed for 1–4 nights (though sometimes much longer) with detectors recording from dusk through dawn. Stationary acoustics were linked with occupancy at the 5 km scale $({q}_{{qt}})$ using a similar observation process as for mobile transects. A Poisson count-detection model for auto IDs was conditional on occurrence at the quad level $({q}_{{qt}})$ instead of abundance at the transect level $\left({M}_{{kt}}\right)$ (Fig. 1-C2, See Supplementary Methods 3).

$${v}_{{qtj}}^{(s)} \sim {{{\rm{Poisson}}}}\left({\delta }_{{qtj}}^{\left(s\right)}\times {q}_{{qt}}+{\omega }_{{qtj}}^{\left(s\right)}\right)$$

This formulation is a false-positive extension of recent multi-scale, ‘continuous-time’ occupancy models^46^ assuming a standard time exposure rate of one per site-night. Thus, the overall detection rate ${\delta }_{{qtj}}^{\left(s\right)}$ is the average encounter rate (i.e., count of true detections per night *j*) given the species was present, which is an implicit combination of local abundances and per-capita detection rates. Then, just as for mobile transects, a subset of manually reviewed ${n}_{{qtj}}^{\left(s\right)}$ and confirmed ${k}_{{qtj}}^{\left(s\right)}$ auto IDs for tricolored bat were included via a hypergeometric formulation to estimate false-positive and true-positive rates (Fig. 1-C2, Supplementary Methods 3). For covariates on ${\delta }_{{qtj}}^{\left(s\right)}$, we included fixed effects of total daily precipitation and minimum air temperature. Day of year effects were considered but removed due to convergence issues. The false-positive rate per night ${\omega }_{{qtj}}^{\left(s\right)}$ was formulated the same as for ${\omega }_{{qtj}}^{\left(m\right)}$, using hierarchically nested random effects of observation night within project ID (Supplementary Methods 3).

### Stationary acoustics (MLE) and live capture data

A small subset of data collected and submitted by USFWS used a statistical decision rule (i.e., ‘MLE method’^56,57^) to remove potential false-positive detections using a p-value threshold of 0.05. For live-capture data and ‘MLE’-protocol stationary acoustics (where suspected false-positives were removed prior to analysis), we specify separate traditional occupancy models for each data type at the 5-km x 5-km scale (Fig. 1-C3 [orange] and Fig. 1-C4 [blue]) given occupancy at the 5-km x 5-km scale $({q}_{{qt}})$ and detection rates (MLE:${p}_{{qtj}}^{\left({mle}\right)}$, Live capture: ${p}_{{qtj}}^{\left({cap}\right)}$). We used minimum daily air temperature, total daily precipitation, and day of year (with fixed linear and quadratic effects on day of year) as predictors for both data types. For live captures, we also included a random intercept by NABat project on the detection probability $\left({p}_{{qtj}}^{\left({cap}\right)}\right)$ to account for differences in sampling methods. Refer to Supplementary Methods 4 for more details.

### Absolute vs relative abundance interpretations from unmarked populations

Given sensitives in the intercept of expected abundance estimates (N and/or λ) to assumption violations (e.g., no unmodeled or mis-specified heterogeneity) in unmarked abundance models, best practices are currently to treat inferences as those pertaining to relative abundance^58–60^ (i.e., covariate effects, proportional trends over time, population growth rates, demographic rates, etc…). Therefore, we interpret our expected abundance estimates as relative rather than absolute. However, similar to prior work^13,61^, we maintain interpretation of the occupancy probability estimates as absolute. This was given the multi-scale nature of the change of support formulation^47^ with additional tunable parameters for transect-level (${\theta }_{k}^{m}$, Fig. 1B) and quadrant-level availability (${\theta }_{{qt}}$, Fig. 1B), and the nature of the complimentary log-log link where occupancy probabilities saturate near one above modest values of expected abundance.

### Data processing and cleaning

Data were accessed from the NABat database using the official data request process^49–51^ for each data type and formatted as long-format encounter histories by location and night. Information on accessing data, data request numbers, data processing and cleaning steps are documented in Supplementary Methods 1. Descriptions of predictive covariates used for relative abundance and local availability (including spatial scale, source, and reasoning for inclusion) are detailed in Supplementary Methods 5 and Supplementary Methods 6.

### Separate vs integrated model comparison

We fitted separate models using data from each individual summer monitoring method, in addition to the fully integrated model, using data from 2016 onward to investigate the improvements to population inferences. The starting year of 2016 was selected to align data sets more fully, given that stationary acoustic data before 2016 was limited (Supplementary Table 1). For the separate analyses of stationary acoustic and live capture data, a logit link was used to model occupancy probability at the 10 km scale instead of the ‘cloglog’ (assumed in the integrated model). A separate model for ‘MLE’ protocol stationary data was not fit due to limited sample sizes.

### Statistics and reproducibility

Statistical analyses were conducted for each model (MS-iSDMs 2012–2022 and 2016–2022, and each independent model type 2016–2022) in JAGS^62^ using R^63^ and the JagsUI^64^ package in a Bayesian framework. MCMC settings of each model are documented in Supplementary Methods 7 (Supplementary Table 3). We used visual assessment of MCMC chains and parameter R-hat^65^ values of less than 1.1 assess convergence of the MCMC chains. JAGS code for the MS-iSDM is provided in Appendix S2 and as part of a US Geological Survey data release^31^. Across all field method types, observations at the site by night level served as temporal replicates for each location and year. Stationary acoustics also typically had 4 spatial replicates per grid cell (1 per quadrant^8^, Fig. 1). The total number of site x night observations for each survey method are provided in Table 1, and for each survey method and year in Supplementary Table 1.

### Model evaluation

Model evaluation is particularly difficult in integrated models given data sets of different qualities and quantities^12^. Because the primary intent of this work was to inform where the species is likely to occur (i.e., predicting occupancy probability across the range) to support conservation management, we evaluated model accuracy using Area Under the Curve (AUC), a measure of (in-sample) classification accuracy that considers classification rates of both presences and absences^66^. We also used a posterior predictive check^67^ to visually assess the models of mobile acoustic and stationary acoustic data by comparing the observed nightly counts to expected values. We also used a leave-out data set (n = 206) of confirmed presences at the quad level as validation data, for which we predicted occupancy probabilities and compared them to those from all sampled locations.

### Predictions from the MS-iSDM across the species range

While data from the entire maternity season informed the MS-iSDM, predictions were focused specifically on the pre-volancy season (May 1–July 15) each year as the primary time period of interest for NABat’s summer status and trends^8,18^. Thus, all species distribution predictions and trend calculations are representative of the pre-volancy season. All continuous covariates were scaled for each grid cell and quadrant in the species range, based on the mean and standard deviations of the available data for each covariate. Values of maximum elevation were clamped at 6 standard deviations greater than the sampled mean (i.e., above the 99.9th percentile of sampled values) to avoid extrapolation beyond the range of observed values. Then, looping over all grid cells in the species range and 5000 MCMC samples for covariates effects on ${\lambda }_{{it}}$ and ${\theta }_{{qt}}$, predictions for expected abundance were made for each grid cell (at the 10-km x 10-km scale) and predictions for the unconditional occupancy probability ${\phi }_{{qt}}$ were made for each quad (5-km x 5-km), year, and MCMC sample. Predictions were summarized for each spatial unit and year by taking the means and 95% credible intervals (lower limit, upper limit, and interval width).

### Status and trends estimates (grid cell and range-wide)

Range-wide, derived population status variables of average relative abundance ${\lambda }_{t}^{{range}}$ and average occupancy probability ${\phi }_{t}^{{range}}$ in the pre-volancy season each year were calculated by taking the mean of predicted ${\lambda }_{{it}}$ and ${\phi }_{{qt}}$ across all spatial units (grid cells and quads respectively) for every MCMC sample and year (as in Udell et al. ^18^). We calculated trends in these derived parameters as the total proportional change in ${\lambda }_{t}^{{range}}$ and ${\phi }_{t}^{{range}}$ between 2012–2022 for each MCMC sample, and summarized the posterior distribution of these trends as the mean, standard deviation, 95% credible intervals, and the probability that the trend was less than one.

### Reporting summary

Further information on research design is available in the Nature Portfolio Reporting Summary linked to this article.

## Supplementary information

Supplementary Information

Reporting Summary

## Data Availability

Data supporting this research are available from North American Bat Monitoring Program (NABat) database^49–51^ [https://sciencebase.usgs.gov/nabat/#/results], with restrictions [including non-disclosure agreements, licensing, other agreements]. The platform is developed and maintained by the US Geological Survey to provide shared, permission-controlled access to scientific data products and resources. Due to sensitivities around bat data, including private land ownership and concern for the safety of vulnerable populations, original data contributors are responsible for managing permissions and data access through the NABat Partner Portal. Users may restrict access to their project-level data, grant access upon request, or make data publicly available. Parties may request access to these data by following steps documented at https://www.nabatmonitoring.org/get-data. The parameters of the dataset drawn from the NABat database, date of the export, and database version are documented in the references^49–51^ and are available on the NABat Data Request Archive (see NABat Request Numbers 166, 167, 172) located at: https://sciencebase.usgs.gov/nabat/#/data/requests/all. Non-sensitive data, model results and predictions, and source data used to make figures are available as a US Geological Survey data release^31^ (10.5066/P1FKYTMA).

## Code availability

Model code has been provided as supplementary material (Appendix S2) and as part of as a US Geological Survey data release^31^ (10.5066/P1FKYTMA).
