# Supplementary material for: Integrated distribution modeling resolves asynchrony between bat population impacts and occupancy trends through latent abundance
Source: Commun Biol. 2025 May 30;8:832. doi: 10.1038/s42003-025-08238-x (PMC12125192; doi:10.1038/s42003-025-08238-x)
Supplement: Supplementary file 1 — Supplementary Information [file 42003_2025_8238_MOESM1_ESM.pdf]

Supplementary materials for: Integrated distribution modeling resolves asynchrony between bat population impacts and occupancy trends through latent abundance

Journal: Communications Biology

Authors: [1]Udell, Bradley J., [2]Stratton, Christian., [3]Irvine, Kathryn M., [1]Straw, Bethany Rose, [4]Reichard, Jonathan D., [5]Gaulke, Sarah, [5]Coleman, Jeremy. T.H., [6]Tousley, Frank C., [1]Schuhmann, Andrea N., [1]Inman, Richard D., [7]Turner, Melinda., [5]Nystrom, Sarah., [1]Reichert, Brian E.

1 United States Geological Survey, Fort Collins Science Center, Fort Collins, Colorado, 80526, USA

2 Department of Mathematical Sciences, Montana State University, Bozeman, MT, 59717, USA

3 United States Geological Survey, Northern Rocky Mountain Science Center, Bozeman, Montana, 59717, USA

4 Colorado Cooperative Fish and Wildlife Research Unit Department of Fish, Wildlife, and Conservation Biology Colorado State University Fort Collins, CO 80523, USA

5 United States Fish and Wildlife Service, Ecological Services, Hadley, Massachusetts, 01035, USA

6 Colorado Natural Heritage Program Colorado State University Fort Collins, CO 80523, USA

7 United States Fish and Wildlife Service, Ecological Services, State College, Pennsylvania, 16801, USA

Disclaimer: "Any use of trade, firm, or product names is for descriptive purposes only and does not imply endorsement by the U.S. Government."

Appendix S1. Supplementary materials and methods

Supplementary Methods 1: Data processing and cleaning

Data supporting this research are available from [North American Bat Monitoring Program (NABat) database, https://sciencebase.usgs.gov/nabat/#/results], with [restrictions, including non-disclosure agreements, licensing, other agreements]. The platform is developed and maintained by the U.S. Geological Survey (USGS) to provide shared, permission-controlled access to scientific data products and resources. Due to sensitivities around bat data including private land ownership and concern for the safety of vulnerable populations, original data contributors are responsible for managing permissions and data access through the NABat

Partner Portal. Users may restrict access to their project-level data or make data publicly available. Parties may request access to these data by following steps documented at https://www.nabatmonitoring.org/get-data. The parameters of the dataset drawn from the NABat database, date of the export and database version are documented in the references[1-3] and are available on the NABat Data Request Archive located at https://sciencebase.usgs.gov/nabat/#/data/requests/all.

All NABat stationary acoustic data records that could be reconciled at the 5km resolution were included in the analysis, while those that could not be reconciled were filtered out. Stationary records could be reconciled at the 5km resolution when one of the following were true: coordinates were provided by the data contribution for the monitored location, or 2) a quadrant (hereafter, “quad”; e.g., “NW”) was including in the location name.

For mobile transect acoustics, all data were summarized and included at the NABat grid cell level. When spatial files were provided for mobile transect locations, a value for observed transect lengths was also derived. When such information was lacking, a proxy transect length was substituted based on the total length of the shortest distance line which connected all point locations (i.e., georeferenced audio files) associated with each transect. These proxy values were provided in the NABat data request, and were calculated in PostGIS by taking the first and last recorded point as the starting and end points, and using Dijkstra Shortest Path Algorithm with a fully connected graph to assign the remaining points position and calculate the resulting length. We filtered out transects with inadequate information or sampling effort, including those which had observed transects less than 100m, or had missing observed transects and predicted transect lengths based on less than 25 points.

Because live-capture data informs occupancy at the NABat quad (5km x 5km), point locations were required to reconcile these data. Thus, capture data submitted to NABat without an associated NABat grid cell were not included.

Supplementary Table 1: Monitoring effort (number of North American Bat Monitoring Program (NABat) grid cells [10km x 10km] and total sampling nights*locations) per monitoring data type each year from 2012 – 2022 for tricolored bats.

Monitoring type	Effort type	2012	2013	2014	2015	2016	2017	2018	2019	2020	2021	2022
Capture	Grid cells	116	194	218	252	338	230	151	134	22	216	264
	Sample nights * locations	817	1,465	1,079	1,502	1,712	929	810	524	77	1245	1,729
Mobile acoustic (with manual review)	Grid cells	61	74	59	77	108	96	51	42	85	64	40
	Sample nights * locations	103	106	85	109	147	130	85	65	116	86	64
Mobile acoustic (without manual review)	Grid cells	487	793	855	735	981	902	964	803	520	619	393
	Sample nights * locations	1,275	2,100	2,203	1,939	2,474	2,439	2,354	1,849	1,279	1,453	913
Stationary acoustic (with manual review)	Grid cells	0	5	1	3	34	66	50	62	85	103	65
	Sample nights * locations	0	35	13	9	310	671	227	920	579	633	322
Stationary acoustic (without manual review)	Grid cells	15	51	110	271	253	308	242	365	424	397	325
	Sample nights * locations	252	1,551	2,889	5,409	9,642	21,863	6,468	9,220	9,700	8,070	6,257
Stationary acoustic (using 'MLE' approach)	Grid cells	0	0	0	2	24	18	35	26	21	6	0
	Sample nights * locations	0	0	0	3	92	114	179	117	73	29	0

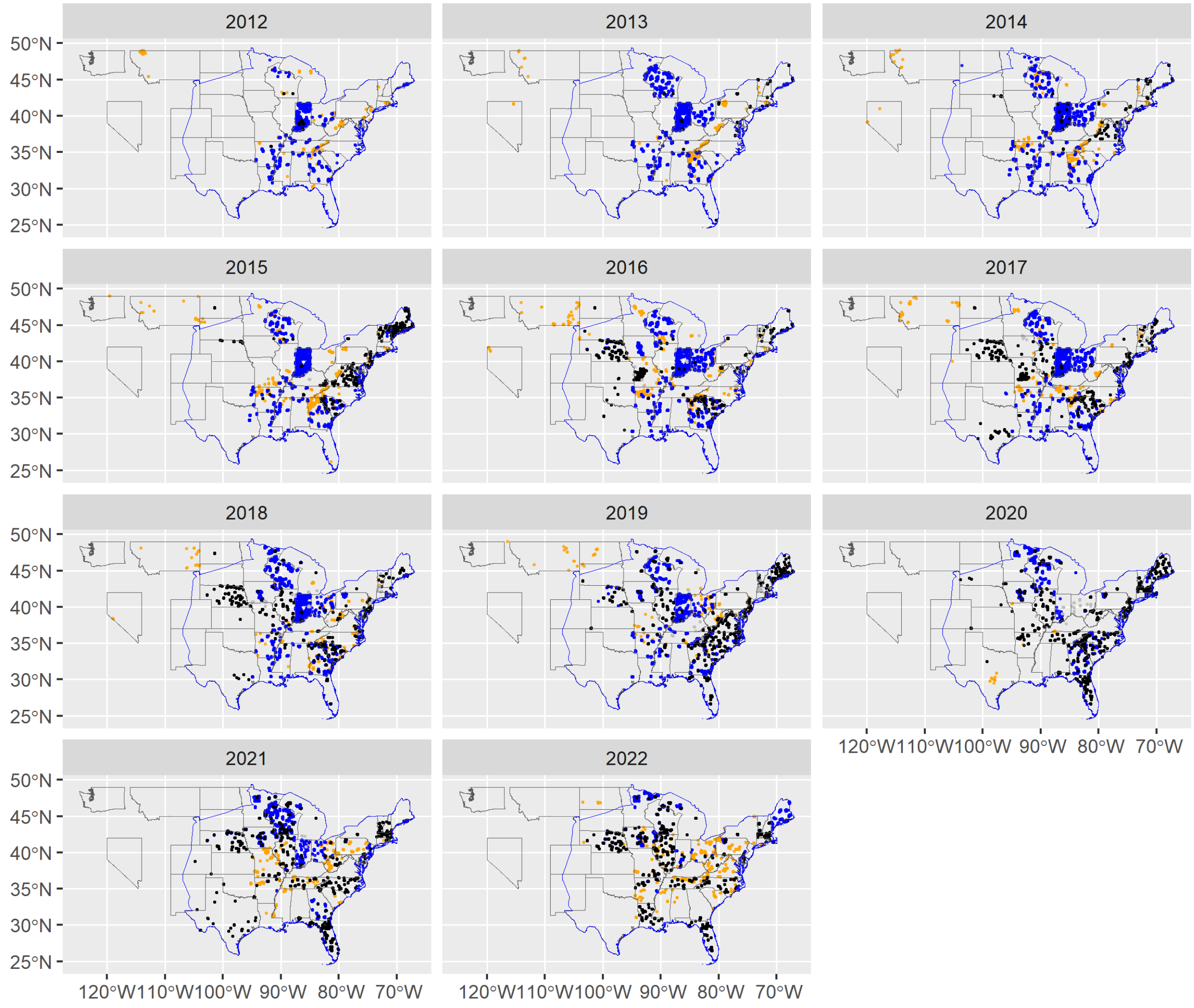

Supplementary Figure 1: Sampling effort for tricolored bats by monitoring type (blue = mobile transects, black = stationary acoustics, grey = stationary acoustic (mle), orange = capture) and year. State polygons are from the Database of Global Administrative Areas[4] (Global Administrative Areas, 2018)

Supplementary Methods 2: Mobile transects observation model

For the mobile transect data, we let $v_{ktj}^{(m)}$ denote the observed number of autoIDs classified as a tricolored bat recording during a nightly visit *j* to a transect *k* during time-period (year x within-summer-season) *t*. The autoIDs are a combination of correctly classified recordings to tricolored bat (denoted as $\mathrm{K}_{ktj}^{(m)}$, which is unknown) that occur at a rate of $\left(\delta_{ktj}^{(m)} \times M_{kt}\right)$ on average, and false positive recordings from non-target sources (denoted as $\mathrm{Q}_{ktj}^{(m)}$, which is also unknown) that occur at a rate of $\omega_{ktj}^{(m)}$ on average. We note that covariates and/or random effects can be incorporated to account for heterogeneity in $\omega_{ktj}^{(m)}$. Then similar to Udell et al. (2024)[5], we specify

$$\left[v_{ktj}^{(m)} | M_{kt}\right] = \mathrm{Poisson}\left(\delta_{ktj}^{(m)} \times M_{kt} + \omega_{ktj}^{(m)}\right)$$

with $\log\left(\delta_{ktj}^{(m)}\right) = w'\alpha$.

As in Udell et al. 2024, we used the manual review formulation from Doser et al. (2021)[6], where the latent number of true tricolored bat detections $\mathrm{K}_{ktj}^{(m)}$ follows a binomial distribution,

$$\left[\mathrm{K}_{ktj}^{(m)} | \, M_{kt}\right] = \mathrm{Binomial}\left(v_{ktj}^{(m)}, \frac{\delta_{ktj}^{(m)} \times M_{kt}}{\delta_{ktj}^{(m)} \times M_{kt} + \omega_{ktj}^{(m)}}\right)$$

and $\frac{\delta_{ktj}^{(m)} \times M_{kt}}{\delta_{ktj}^{(m)} \times M_{kt} + \omega_{ktj}^{(m)}}$ is the true positive rate of the autoIDs . The number of incorrectly classified recordings as tricolored bat is then given by $\mathrm{Q}_{ktj}^{(m)} = \mathrm{v}_{ktj}^{(m)} - \mathrm{K}_{ktj}^{(m)}$.

The manually reviewed recordings allow for estimating the number of detected and correctly classified recordings to tricolored bat $\left(\mathrm{K}_{ktj}^{(m)}\right)$. For a given mobile transect survey on night *j* within transect *k* and time period *t*, we have a set number, $\mathrm{n}_{ktj}^{(m)}$ of reviewed recordings, and of those some number that were confirmed to be a tricolored bat recording, denoted $\mathrm{k}_{ktj}^{(m)}$. Then, as proposed originally in Chambert et al. (2018)[7], we specified the following for the manual review portion of the observation process:

$$\left[\mathrm{k}_{ktj}^{(m)} \mid \mathrm{n}_{ktj}^{(m)}, \mathrm{K}_{ktj}^{(m)}\right] = \mathrm{Hypergeometric}\left(\mathrm{n}_{ktj}^{(m)}, \mathrm{k}_{ktj}^{(m)}, K_{ktj}^{(m)}, \mathrm{Q}_{ktj}^{(m)}\right)$$

Accounting for heterogeneity in the average per-individual detection rate

Following NABat guidance[8], our focus for inference was on the pre-volancy season (May 1 – July 15); however, we also included data through mid-August (prior to the start of fall migration). As described in the main text, we modeled the difference in abundance pre- and post-volancy by including an indicator variable β_{post}in the linear predictor of λ_{it} and by allowing the population M_{kt} to vary before and after July 15 each year. To account for difference in per-capita activity rates, we used a quadratic relationship on day of year (paired with a different abundance pre- and post- volancy) to model this general trend while remaining flexible enough to reflect linear relationships if quadratic was not supported. Because of seasonal and environmental differences in bat activity between locations, we allowed the quadratic effect to have random slopes by transect *k*:

$$\delta_{kjt}^{(m)} = \delta_0 + \delta_{1_k} * doy_{kj} + \delta_{2_k} * doy_{kj}^2$$

$$\delta_0 \sim \mathrm{Norm}(0, \tau = 0.1)$$

We constrained the random effect for the quadratic term of day of year to be strictly negative (downward shaped hump) as follows:

$$\delta_{1_k} \sim \text{Norm}(\mu_{d1}, sd_{d1})$$

$$\log\left(\delta_{2_k}\right) \sim \text{Norm}(\mu_{d2}, sd_{d2})$$

We used diffuse Normal(0, τ =0.01) priors for the hyper-means, and weakly informative priors for the standard deviation of these random effects assuming a half-t distribution with scale = 1 and df = 10 (δ_1), and scale = 1 and df = 100 (δ_2). We also bounded the random effect of δ_{2_k} between -5 and 5 on the log scale to avoid extreme values and improve estimation.

Accounting for variability in the false positive rate

To account for heterogeneity in the false positive rate ω_{ktj}^{m}, we included observation level random effects nested within project *k* level random effects:

$$\omega_{proj}^{m} \sim \text{Normal}\left(F_{\mu}^{m},\ \sigma_{proj}^{m}\right)$$

$$\log\left(\omega_{ktj}^{m}\right) \sim \text{Normal}\left(\omega_{proj[ktj]}^{m},\ \sigma_{obs}^{m}\right)$$

We used diffuse normal priors for F_{μ}^{m} and weakly informative half- T priors for the standard deviations (σ_{proj}^{m}: scale = 1, df = 4, $s\sigma_{obs}^{m}$: scale = 1, df = 4).

Supplementary Methods 3*:* Stationary acoustics observation model (with a subset of manual review)

The stationary ARU (automated recording unit) observation process is related to the mobile; however, given high incidence of detecting individuals more than once and the degree of clustering within individuals, we conservatively only make direct inferences to occupancy

instead of abundance, which also provides information on abundance via the probability of zero. Thus, instead of a N-mixture model with a Poisson count-detection process, we specify an occupancy model with a Poisson count detection process, given the latent binary state of local occurrence within a quad q and time period t (q_{qt}). To distinguish the stationary ARU counts $v_{qtj}^{(s)}$ as distinct from the mobile counts $v_{ktj}^{(m)}$, we use the super-script (s) versus (m), as follows:

$$\left[v_{qtj}^{(s)} | q_{qt}\right] = \text{Poisson}\left(\delta_{qtj}^{(s)} \times q_{qt} + \omega_{ktj}^{(s)}\right)$$

$$\left[\mathrm{K}_{qtj}^{(s)} | \, q_{qt}\right] = \text{Binomial}\left(v_{qtj}^{(s)}, \frac{\delta_{qtj}^{(s)} \times q_{qt}}{\delta_{qtj}^{(s)} \times q_{qt} + \omega_{ktj}^{(s)}}\right)$$

$$\left[\mathrm{k}_{qtj}^{(s)} \mid \mathrm{n}_{qtj}^{(s)}, \mathrm{K}_{qtj}^{(s)}\right] = \text{Hypergeometric}\left(\mathrm{n}_{qtj}^{(s)}, \mathrm{k}_{qtj}^{(s)}, \mathrm{K}_{qtj}^{(s)}, \mathrm{Q}_{qtj}^{(s)}\right)$$

Accounting for heterogeneity in the average detection rate

The average detection rate $\delta_{qtj}^{(s)}$ corresponds to the expected count of autoIDs per night of an occupied quad, which differs from $\delta_{ktj}^{(m)}$, the ‘per-individual’ detection rate of the mobile transect observation model. Thus, $\delta_{qtj}^{(s)}$ is an implicit combination of both per-individual detection rates and the expected abundance in each quad. Nightly level covariates of total precipitation and minimum temperature were included to account for heterogeneity, while day of year effects were initially considered but not included due to convergence issues.

Accounting for variability in the false positive rate

In order to account for heterogeneity in the false positive rate ω_{qtj}^{s}, we included observation level random effects nested within project level random effects:

$$\omega_{proj}^{(s)} \sim \text{Normal}\left(F_{\mu}^{s},\ \sigma_{proj}^{s}\right)$$

$$\log\left(\omega_{qtj}^{(s)}\right) \sim \text{Normal}\left(\omega_{proj[qtj]}^{(s)},\ \sigma_{obs}^{(s)}\right)$$

We used diffuse normal priors for F_{μ}^{s}, and weakly informative half-T priors for the standard deviations ($\sigma_{proj}^{(s)}$scale = 1, df = 4, σ_{obs}^{s}: scale = 1, df = 4).

Supplementary Methods 4: Live capture and stationary acoustics (MLE) observation models

Finally, to integrate the "maximum likelihood estimator" (MLE) stationary acoustics from U.S. Fish and Wildlife Service, we assume they are available at the finer grain size of a quad *q* and specify traditional model that accounts for only false negatives. A false negative would be a failure to meet the MLE p-value threshold or no capture record during time-period *t* even if tricolored bat occurred within the quad *q* and time period *t*. We keep the capture and MLE-based data streams separated as two separate observation models. We denote $y_{qtj}^{(cap)}$ as an indicator for whether or not a tricolored bat was captured on visit *j* to quad *q* and time period *t*. We let $y_{qtj}^{(mle)}$represent the MLE based detection/non-detection data. If the MLE p-value < 0.05, tricolored bat was considered detection, and otherwise was considered not detected. To account for the potential false negatives (i.e., traditional occupancy model), we specify the following:

$$\left[y_{qtj}^{(cap)} | q_{qt}\right] = \text{Bernoulli}\left(p_{qtj}^{(cap)} \times q_{qt}\right)$$

$$\left[y_{qtj}^{(mle)} | q_{qt}\right] = \text{Bernoulli}\left(p_{qtj}^{(mle)} \times q_{qt}\right)$$

Both the detection parameters $p_{qtj}^{(cap)}$ and $p_{qtj}^{(mle)}$ were adjusted for heterogeneity across quads, visits, and years using a logit-link function. A random intercept for the NABat data contributor was also included in the link function for the detection rate of capture data.

Supplementary Methods 5: Predictors of tricolored abundance (10km x 10km) and occupancy (5km x 5km).

Supplementary Table 2. Spatial covariates used as predictors of tricolored bat abundance λ at the 10km grid level, and availability θ at the 5km quadrant level given occupancy at the 10km level.

Variable	Covariate	Spatial scale	Reasoning	Source
λ<br>θ	Culvert count (log plus 1)	10km grid cell<br>5km quadrant	Culverts are commonly used by tricolored bat as winter and summer roosts. We expected a positive relationship.	National Bridge Inventory
λ<br>θ	Maximum elevation with quadratic effect	10km grid cell<br>5km quadrant	Elevation is a known consideration for most bats, and quadratic effects can be important. We expect a convex quadratic relationship as in Udell et al. 2024.	USGS GTOPO 30
λ<br>θ	Conifer forest cover	10km grid cell<br>5km quadrant	Forest cover is important for summer roost and foraging habitat. Differences by forest types are known considerations. We expected a positive relationship.	USDA Forest Service - Forest Inventory and Analysis (FIA) Program & Remote Sensing Applications Center
λ<br>θ	Oak forest cover	10km grid cell<br>5km quadrant	Forest cover is important for summer roost and foraging habitat. Differences by forest types are known considerations. We expected a positive relationship.	USDA Forest Service - Forest Inventory and Analysis (FIA) Program & Remote Sensing Applications Center
λ<br>θ	Deciduous (non-oak)<br>forest cover	10km grid cell<br>5km quadrant	Forest cover is important for summer roost and foraging habitat. Differences by forest types are known considerations. We expected a positive relationship.	USDA Forest Service - Forest Inventory and Analysis (FIA) Program & Remote Sensing Applications Center
λ<br>θ	Wetlands cover with quadratic effect	10km grid cell<br>5km quadrant	Wetlands have an abundance of water and insect prey. Expect highest abundances at intermediate levels of cover.	MODIS (250m)
λ<br>θ	Physiographic diversity	10km grid cell<br>5km quadrant	Related to plant diversity, topographic roughness, and habitat complexity. Expect a positive relationship.	Theobald et al. (2015)[9]

Processing forest types

Given the inherent correlations of percent landcover types (i.e., because their values sum to 1), and limitations of analysis when predictors are highly correlated, we processed the raw data layers into combined groups to aid analysis. First, we combined percent dominant oak cover and percent subdominant oak cover into a single group (percent oak cover). Next, because percent oak cover and percent deciduous forest cover were highly correlated, we subtracted percent oak cover from percent deciduous forest cover in each cell (percent deciduous - percent oak cover), to remove correlations between these variables. Then, percent total oak, percent deciduous (non-oak), and percent coniferous forest were included as three separate predictors in the model at both the 10km scale and 5km scale.

Supplementary Methods 6: Integrating winter colony counts: winter-to-summer metapopulation connectivity

We included a winter-to-summer connectivity metric as a spatiotemporal predictor of abundance for each grid cell and year. This connectivity metric linked summer and winter populations based on the species seasonal migration behavior[5]. Specifically, it linked the annual (based on year in January) population abundance in winter hibernacula recorded in the NABat database from a separate status and trends analysis[10] with expected abundance in the summer distribution. Following Udell et al. (2024)[5], we calculated a ‘potential metapopulation connectivity metric’[11] that predicted the relative number of summer migrants to each grid cell based on: 1) the abundance of bats in the prior winter each year in each known hibernaculum, and 2) the probability that winter and summer locations were connected based on a seasonal migration kernel with one parameter (average seasonal migration distance) and the distance between each documented hibernaculum and grid cell. This metric provides a potentially useful spatial predictor of bat summer populations and also a way of linking regional trends between

winter and summer populations. For example, winter abundances of tricolored bats have declined drastically since the arrival of white-nose syndrome (WNS), with regional differences depending on the timing of WNS arrival[12]. Thus, this metric also captured the potential spatiotemporal influence of WNS impacts in known winter populations on the summer abundance distribution.

Using an exponential dispersal kernel with the mean winter-to-summer migration distance (α = inverse migration distance), the probability (p_{iw}) that a summer grid cell *i* and winter hibernaculum *w* were connected was based on the distance between them (d_{iw}):

$$p_{iw} = \exp(-\alpha \times d_{iw})$$

These p_{iw} were then scaled to sum to one for each hibernacula *j* by taking $p_{iw} = \frac{p_{iw}}{ptot_w}$, where $ptot_w$ was the sum across all p_{iw} for each hibernaculum *w*. This redefined p_{iw} as the relative probability of seasonal connectivity of hibernaculum *w* to each summer grid cell i. The pairwise connectivity each year *y*, between each grid cell *i* and known winter hibernaculum *w*, was then calculated by multiplying p_{iw} by hibernaculum abundances each year $\left(A_{wy}\right)$.

$$S_{iwy} = p_{iw} \times A_{wy}$$

Thus, S_{iwy} was a proxy for the relative number of seasonal migrants between locations, and because p_{iw} sums to one for each hibernaculum, the sum of all seasonal migrants from hibernaculum *w* each year was equal to the hibernaculum abundance in the same year: $\sum_i S_{iwy} = A_{wy}$. Finally, the total seasonal connectivity in each grid cell and year was calculated by summing the contributions from all hibernacula to each summer grid cell:

$$S_{iy} = \sum_w S_{iwy}$$

The average winter-to-summer migration distances for tricolored bat reported in the literature was 101.1km (n = 6), where citations are available in Udell et al. (2024)[5].

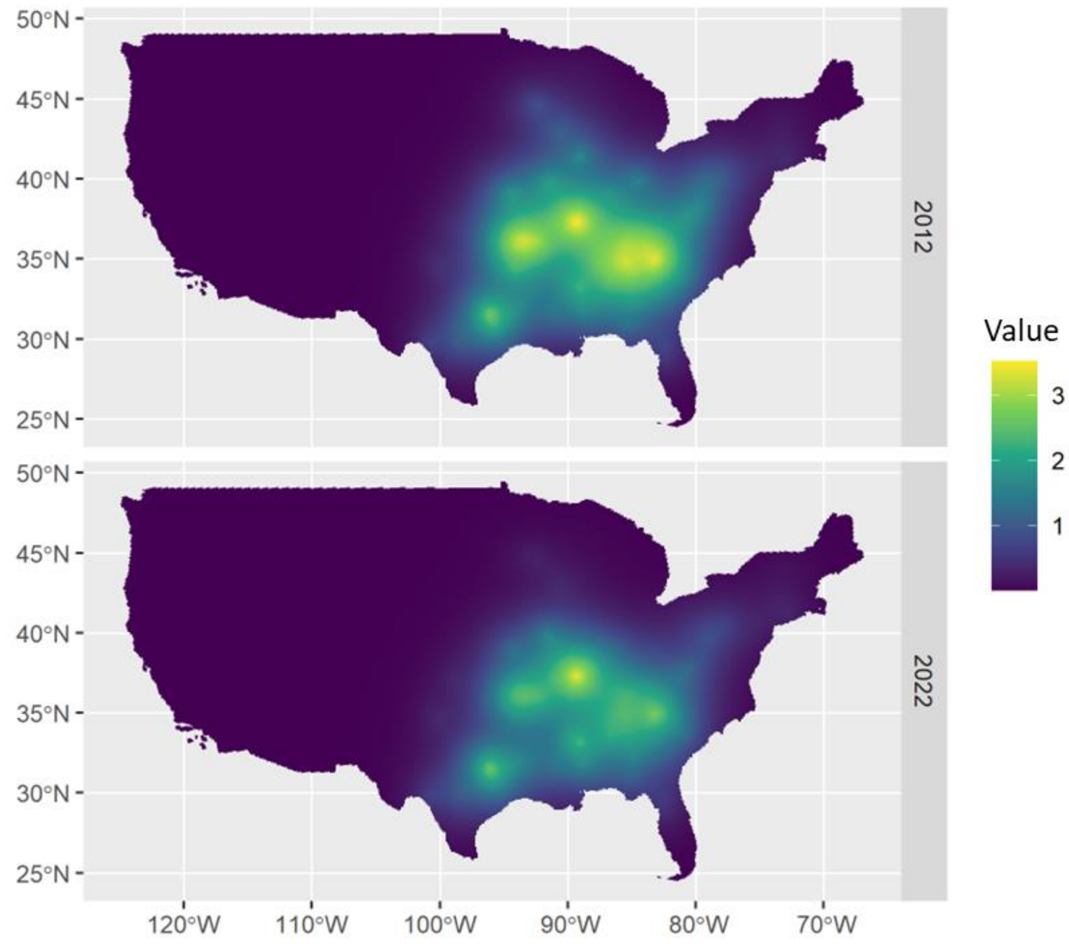

Supplementary Figure 2: Winter-to-summer population connectivity for tricolored bats in 2012 and 2022, depicting values on a ‘log plus one’ scale for visualization purposes. Declines in values between 2012-2022 represent population declines due to white nose-syndrome, and a reduction in the expected number of seasonal migrants to grid cells in the summer.

Supplementary Methods 7: Model fitting

We fitted the multi-scale integrated species distribution model MS-iSDM model and each individual model in JAGS[13] using R[14] and the JagsUI[15] packages using Bayesian inference. Visual assessment of Markov chain Monte Carlo (MCMC) chains and parameter R-hat values of less than 1.1 ensured that MCMC chains converged[16].

Supplementary Table 3. Markov chain Monte Carlo (MCMC) setting in JAGS for fitting each species distribution model for tricolored bat. MS-iSDM = multi-scale integrated species distribution model.

Model	Iterations	Adapt	Burn in	Chains
MS-iSDM-2012	90,000	40,000	25,000	8
MS-iSDM-2016	260,000	40,000	100,000	3
Mobile only	80,000	40,000	20,000	8
Stationary only	80,000	40,000	20,000	8
Capture only	80,000	40,000	20,000	8

Appendix S2: Supplementary JAGS code for the MS-iSDM

Disclaimer: This software has been approved for release by the U.S. Geological Survey (USGS). Although the software has been subjected to rigorous review, the USGS reserves the right to update the software as needed pursuant to further analysis and review. No warranty, expressed or implied, is made by the USGS or the U.S. Government as to the functionality of the software and related material nor shall the fact of release constitute any such warranty. Furthermore, the software is released on condition that neither the USGS nor the U.S. Government shall be held liable for any damages resulting from its authorized or unauthorized use

JAGS code (saved as .txt) for the multi-scale iSDM model

```
#################################################################
# Description: this code creates a JAGS model file (saved as MSISDM.txt)
# used to fit the multi-scale integrated species distribution model (MS-iSDM)
# from the manuscript. Alternatively, the model can be created outside of R
# by copying all text within and including “model{}” (lines 33 – 314), and
# pasting it in a .txt editor. All code wrapped within model{} constitutes
# the JAGS model file, while the cat() function creates writes the JAGS model
# as a .txt file.

# Code creation date: 11/20/2023

# Citation of manuscript: Integrated distribution modeling resolves
# asynchrony between bat population impacts and occupancy trends through
# latent abundance. Udell, Bradley J., Stratton, Christian., Irvine, Kathryn
# M., Straw, Bethany Rose, Reichard, Jonathan D., Gaulke, Sarah, Coleman,
# Jeremy. T.H., Tousley, Frank C., Schuhmann, Andrea N., Inman, Richard D.,
# Turner, Melinda., Nystrom, Sarah., Reichert, Brian E. 2025. Communications
Biology.

# Code was written in program R, version 4.1.2 (2021-11-01) and creates a
# model file for JAGS version 4.3.

# Disclaimer: This software has been approved for release by the U.S.
# Geological Survey (USGS). Although the software has been subjected to
# rigorous review, the USGS reserves the right to update the software as
# needed pursuant to further analysis and review. No warranty, expressed or
# implied, is made by the USGS or the U.S. Government as to the functionality
# of the software and related material nor shall the fact of release
# constitute any such warranty. Furthermore, the software is released on
# condition that neither the USGS nor the U.S. Government shall be held
# liable for any damages resulting from its authorized or unauthorized use

########################################
```

```
cat("

model{
  ########################################
  ### ecological process ###
  ########################################

  ## time component - first order autoregressive process (AR1)
  sigma2_t <- pow(sigma_t, 2)
  for(i in 1:nyears){
    mu_t[i] <- mu_b
    Sigma_t[i,i] <- sigma2_t + const
    for(j in (i+1):nyears){
      Sigma_t[i,j] <- sigma2_t * pow(rho, dist_time[i,j])
      Sigma_t[j,i] <- Sigma_t[i,j]
    }
  }
  Sigma_t_inv <- inverse(Sigma_t)
  b ~ dmnorm(mu_t, Sigma_t_inv)
  mu_b ~ dnorm(0,0.1)
  rho ~ dbeta(3, 1)
  sigma_t ~ dt(0, 1, 1) T(0, 1e2)

  ## 10km x 10km cell level abundance and occupancy
  for(row in 1:nrow_grts){
    # grid level abundance
    log(lambda_grid[row]) <- inprod(X[row,1:p_lambda], beta[1:p_lambda]) +
      b[grts_row_to_year[row]]

    N[row] ~ dpois(lambda_grid[row])

    # site level occupancy
    z[row] <- N[row] > 0
  }

  ## multiscale transect level abundance
  for(row in 1:n_transect_yp){
    #sampling exposure rate of transect within grid cell
    log(pthin[row]) = -beta_tl* tl_recip[transect_from_tranYP[row]]

    #transect level abundance conditional on grid cell level abundance
    M[row] ~ dbin(pthin[row], N[grts_year_post_id[row]])
  }

  ## multiscale 5km x 5km level occurrence
  for(row in 1:nrow_subsites){
    # subsite (quadrant) level availability
    logit(theta[row]) <- inprod(W[row, 1:p_theta], alpha[1:p_theta])

    # subsite (quadrant) level occurrence conditional on grid cell occupancy
```

```
    q[row] ~ dbern(z[subsite_to_site_ndx[row]] * theta[row])
  }

  ########################################
  ### likelihood - detection processes ###
  ########################################
  ## stationary data - unconfirmed
  for(row in 1:nrow_stationary_unconf){
    # auto id process
    stationary_unconf_auto_mean[row] <- q[stationary_unconf_night_to_subsite_
ndx[row]] *
      stationary_unconf_mu[row] + stationary_omega_uc[row]
    stationary_unconf_autos[row] ~ dpois(stationary_unconf_auto_mean[row])
    # drop cols 2 and 3 to remove covariates not used (DOY and DOY^2)
    log(stationary_unconf_mu[row]) <- inprod(
      V_stationary_unconf_mu[row, c(1, 4:p_stationary_mu)],
      delta_stationary_mu[c(1, 4:p_stationary_mu)]
      )

    #observation within project random effect on omega stationary
    log(stationary_omega_uc[row]) <- sa_uc_omega_obs[row]
    sa_uc_omega_obs[row] ~ dnorm(sa_proj_omega[saproj_unconf[row]], tau_sa_ob
s_omega)
  }

  ## stationary data - confirmed
  for(row in 1:nrow_stationary_conf){
    # subsite-night level mu (detection rate) and omega (for stationary)

    # drop cols 2 and 3 to remove covariates not used (DOY and DOY^2)
    log(stationary_conf_mu[row]) <- inprod(
      V_stationary_conf_mu[row, c(1, 4:p_stationary_mu)],
      delta_stationary_mu[c(1, 4:p_stationary_mu)]
    )

    # observation within project RE for Omega
    sa_c_omega_obs[row] ~ dnorm(sa_proj_omega[saproj_conf[row]], tau_sa_obs_o
mega)
    log(stationary_omega_c[row]) <- sa_c_omega_obs[row]

    # auto id process
    stationary_conf_auto_mean[row] <- q[stationary_conf_night_to_subsite_ndx[
row]] *
      stationary_conf_mu[row] +
      stationary_omega_c[row]
    stationary_conf_autos[row] ~ dpois(stationary_conf_auto_mean[row])

    # review process
    stationary_tp[row] <- (q[stationary_conf_night_to_subsite_ndx[row]] *
                             stationary_conf_mu[row]) /
```

```
            (q[stationary_conf_night_to_subsite_ndx[row]] *
                                      stationary_conf_mu[row] +
                                      stationary_omega_c[row])
    stationary_K[row] ~ dbinom(stationary_tp[row], stationary_conf_autos[row]
)
    stationary_Q[row] <- stationary_conf_autos[row] - stationary_K[row]
    stationary_nconfirm[row] ~ dhyper(stationary_K[row], stationary_Q[row],
                                          stationary_nreview[row], 1)

  }

  ## mobile data - unconfirmed
  for(row in 1:nrow_mobile_unconf){
    # mean encounter rate for mobile acoustics
    # imposing structural assumptions regarding quadratic effects
    # cols 1 and 2 dropped from matrix below as int and DOY REs hard coded
    log(mobile_unconf_delta[row]) <-
      ma_delta0  +
      ma_delta1[mobile_unconf_tr_id[row]]  * V_mobile_unconf[row, 1] -
      ma_delta2[mobile_unconf_tr_id[row]]  * V_mobile_unconf[row, 2] +
      inprod(
        V_mobile_unconf[row, 3:p_mobile_delta], alpha_mobile_delta[3:p_mobile
_delta]
      )

    # obs within project random effect
    ma_uc_omega_obs[row] ~ dnorm(ma_proj_omega[maproj_unconf[row]], tau_ma_ob
s_omega)
    log(mobile_omega_uc[row]) <- ma_uc_omega_obs[row]

    # auto id process
    mobile_unconf_auto_mean[row] <- mobile_unconf_delta[row] *
      M[mobile_unconf_night_to_site[row]] +
      mobile_omega_uc[row]
    mobile_unconf_autos[row] ~ dpois(mobile_unconf_auto_mean[row])
  }
  ## mobile data - confirmed
  for(row in 1:nrow_mobile_conf){
    # mean encounter rate for mobile acoustics
    # imposing structural assumptions regarding quadratic effects
    # cols 1 and 2 dropped from matrix below as int and DOY REs hard coded
    log(mobile_conf_delta[row]) <-
      ma_delta0 +
      ma_delta1[mobile_conf_tr_id[row]]  * V_mobile_conf[row, 1] -
      ma_delta2[mobile_conf_tr_id[row]]  * V_mobile_conf[row, 2] +
      inprod(
        V_mobile_conf[row, 3:p_mobile_delta], alpha_mobile_delta[3:p_mobile_d
elta]
      )
```

```
    # obs within project random effect
    log(mobile_omega_c[row]) <- ma_c_omega_obs[row]
    ma_c_omega_obs[row] ~ dnorm(ma_proj_omega[maproj_conf[row]], tau_ma_obs_o
mega)

    # auto id process
    mobile_conf_auto_mean[row] <- mobile_conf_delta[row] *
      M[mobile_conf_night_to_site[row]] +
      mobile_omega_c[row]
    mobile_conf_autos[row] ~ dpois(mobile_conf_auto_mean[row])

    # manual review process
    mobile_tp[row] <- (mobile_conf_delta[row] * M[mobile_conf_night_to_site[r
ow]]) /
      (mobile_conf_delta[row] * M[mobile_conf_night_to_site[row]] + mobile_om
ega_c[row])
    mobile_K[row] ~ dbinom(mobile_tp[row], mobile_conf_autos[row])
    mobile_Q[row] <- mobile_conf_autos[row] - mobile_K[row]
    mobile_nconfirm[row] ~ dhyper(mobile_K[row], mobile_Q[row], mobile_nrevie
w[row], 1)
  }

  ## live-capture
  for(row in 1:nrow_capture){
    capture_y[row] ~ dbern(q[capture_night_to_subsite_ndx[row]] * p_capture[r
ow])

    # detection probabilities
    # col 1 dropped from matrix below as intercept is encoded as RE
    logit(p_capture[row]) <- cap_delta0[cap_proj_id[row]]  +
      inprod(
        V_capture[row, 2:ncoefs_capture],  delta_capture[2:ncoefs_capture]
      )
  }
  ## “MLE” (maximum-likelihood estimator) stationary acoustics
  for(row in 1:nrow_fws){
    fws_y[row] ~ dbern(q[fws_night_to_subsite_ndx[row]] * p_fws[row])

    logit(p_fws[row]) <- inprod(
      V_fws[row, 1:ncoefs_fws],  delta_fws[1:ncoefs_fws]
    )
  }
  ######################
  ### Random effects ##
  ######################
  ## stationary project level random effects of false-positive rates
  sd_sa_proj_omega <- 1/sqrt(tau_sa_proj_omega)
  tau_sa_proj_omega ~ dscaled.gamma(1,4)
```

```
  for(i in 1:total_saproj){
    sa_proj_omega[i] ~ dnorm(mu_stationary_omega ,tau_sa_proj_omega)
  }
  mu_stationary_omega ~ dnorm(0, .1)

  ## mobile transect random effects
  ## project level random effects on false-positive rates
  sd_ma_proj_omega <- 1/sqrt(tau_ma_proj_omega)
  tau_ma_proj_omega ~ dscaled.gamma(1,4)
  for(i in 1:total_maproj){
    ma_proj_omega[i] ~ dnorm(alpha_mobile_omega ,tau_ma_proj_omega)
  }
  #mobile transect level random effects of detection rates (day of year effec
ts, DOY )
  for(i in 1:n_transect){
    #random quadratic effect of DOY of average detection rate
    ma_delta1[i] ~ dnorm(ma_mu_d1,ma_tau_d1) # random slope for DOY linear ef
fect
    log(ma_delta2[i]) =  ma_delta2_part[i] # bound random quadratic slope of
DOY to be
    # positive here, so later it can be bound as negative/concave)
    ma_delta2_part[i] ~ dnorm(ma_mu_d2,ma_tau_d2)  T(-5,5) # random slope for
quadratic
    # exclude extreme values to improve convergence
  }

  ## hyper -parms for linear DOY effect on average detection rate
  ma_mu_d1 ~ dnorm(0,0.1)
  ma_tau_d1 ~ dscaled.gamma(1,10)
  ma_sd_d1 = 1 / sqrt(ma_tau_d1)

  ## hyper -parms for quadratic DOY effect on average detection rate
  ma_mu_d2 ~ dnorm(0,0.1)
  ma_tau_d2 ~ dscaled.gamma(1,100)
  ma_sd_d2 = 1 / sqrt(ma_tau_d2)

  ## capture project level random effects for intercepts and DOY slopes on de
tection rates
  for(i in 1:total_capproj){
    cap_delta0[i] ~ dnorm(mu_detla0_cap,tau_detla0_cap)
  }

  ## hyperparms
  mu_detla0_cap ~ dnorm(0, 0.1)
  sd_detla0_cap <- 1/sqrt(tau_detla0_cap)
  tau_detla0_cap ~ dscaled.gamma(1,4)
  ## intercept for average detection per individual
  ma_delta0 ~ dnorm(0, 0.1)

  ##############
```

```
  ### priors ###
  ##############
  ## abundance covariates effects (10km grid cell)
  for(i in beta_inds){
   beta[i] ~ ddexp(0.0, sqrt(2.0)) #independent Laplace priors
  }
  beta[post_ind] ~ dnorm(0, .1)T(0,) #post volancy
  beta[connect_ind] ~ dnorm(0, .1)T(0,) # connectivity
  ## covariates effects for local availability given occupancy at grid cell
  alpha[1] ~ dnorm(0, .1)
  for(i in 2:p_theta){
    alpha[i] ~ ddexp(0.0, sqrt(2.0)) #indepedent Laplace priors
  }
  ## covariate effects for detection rates
  for(i in 1:p_stationary_mu){
    delta_stationary_mu[i] ~ dnorm(0, .01) #stationary acoustic
   # values at indices 2 and 3 not interpreted, but drawn from priors
   }
  for(i in 1:ncoefs_capture){
    delta_capture[i] ~ dnorm(0, .01) #capture
   # values at index 1 not interpreted, but drawn from priors
   }
  for(i in 1:ncoefs_fws){
    delta_fws[i] ~ dnorm(0, .01) # MLE stationary acoustic
   }
  for(i in 1:p_mobile_delta){
    alpha_mobile_delta[i] ~ dnorm(0, .01) #mobile acoustic
   # values at indices 1 and 2 not interpreted, but drawn from priors
   }
  alpha_mobile_omega ~ dnorm(0, .1)

  ## variance components from random effects described in likelihood statemen
ts
  sd_sa_obs_omega <- 1/sqrt(tau_sa_obs_omega)
  tau_sa_obs_omega ~ dscaled.gamma(1,4)

  sd_ma_obs_omega <- 1/sqrt(tau_ma_obs_omega)
  tau_ma_obs_omega ~ dscaled.gamma(1,4)

  ## inverse transect length effect (phi|TL)
  beta_tl ~ dnorm(0, .001) T(0,)
}

", file = "MSISDM.txt")
```

Appendix S3: Supplementary Results

Supplementary Results 1: Supplementary results from the full multi-scale integrated species distribution model MS-iSDM-2012

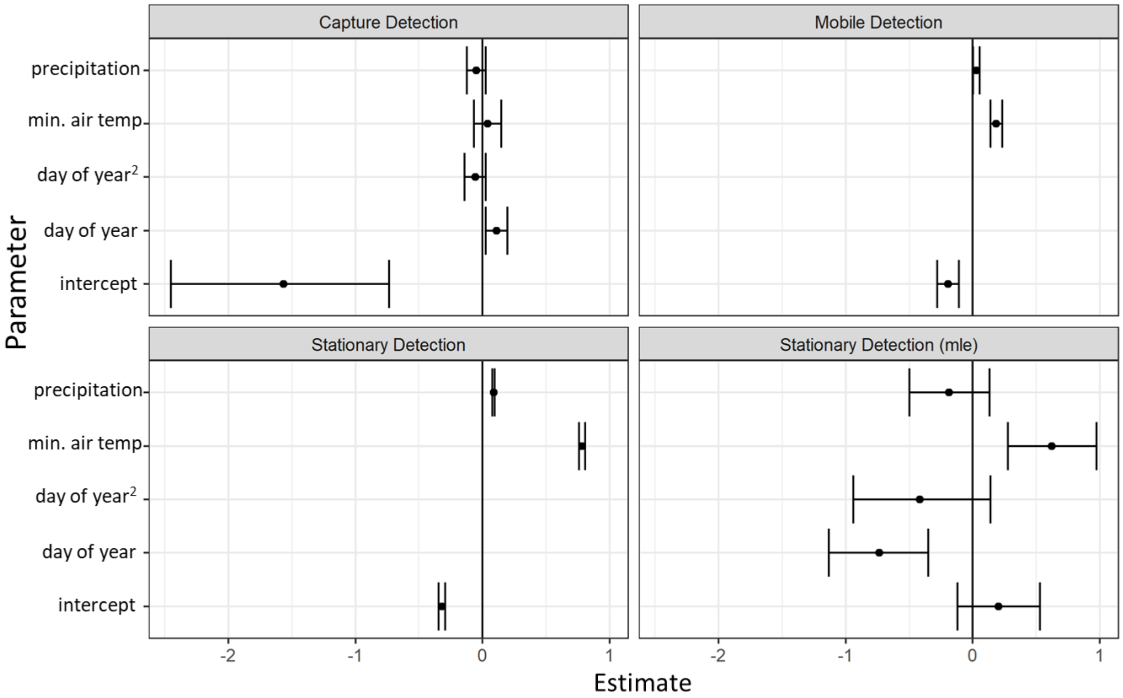

Supplementary Figure 3: Covariate effects on detection probabilities of each monitoring data type for tricolored bats. Note the intercept for capture data is the hyper-mean of a random effect (which reflects the higher uncertainty compared to other data types), where all other intercepts are fixed. The hyper-means of the day of year random slopes for mobile transects are not depicted here, and instead the relationship is depicted below in Supplementary Figure 4. Error bars depict 95% Bayesian credible intervals.

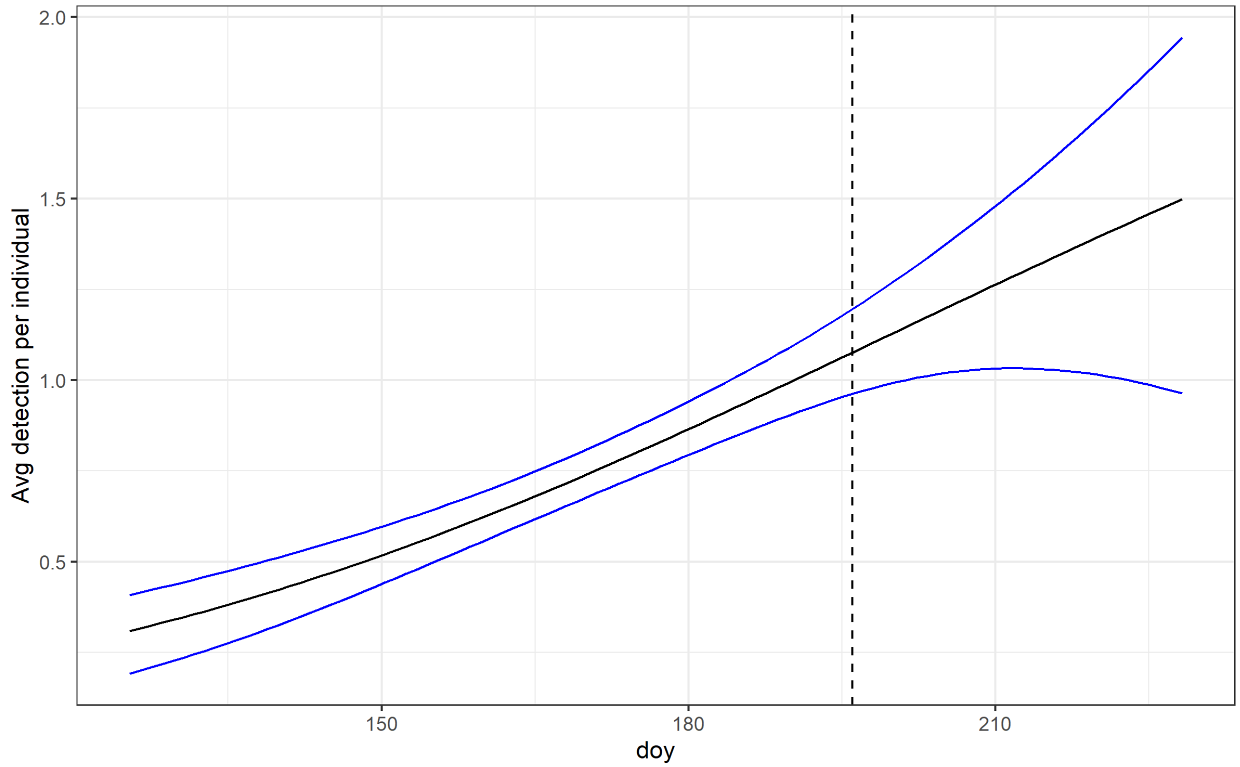

Supplementary Figure 4: Average bat detection probability (using hyper-means of estimated random effects) per individual in M_{kt} (those with home ranges which lie along a transect) for mobile transect acoustics given the day of year. Dashed reference line at day 196 (July 15th) represents the cut off between pre- and post-volancy seasons. Blue lines depict 95% Bayesian credible intervals.

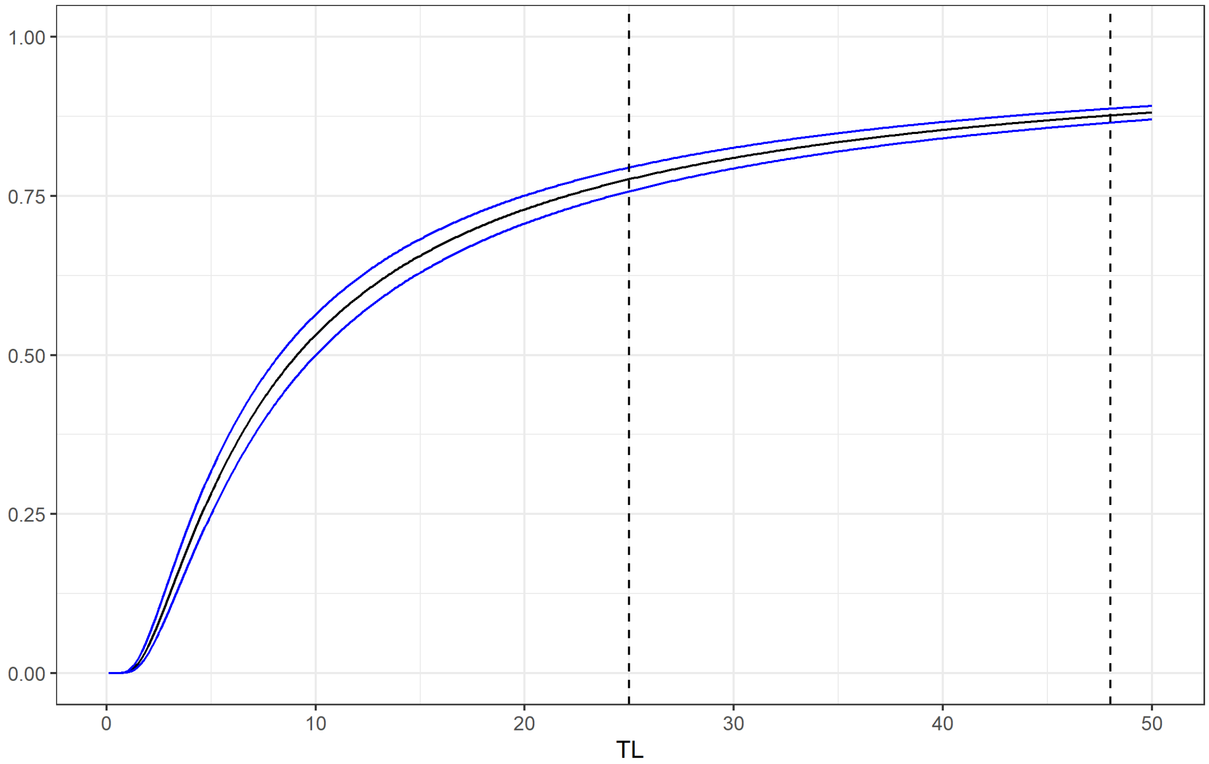

Supplementary Figure 5: Estimated relationship between sampling exposure (average proportion of bats in a grid cell exposed to sampling) and transect length given transect length driven. Dashed reference line 25km and 48km represent the suggested transect lengths in the North American Bat Monitoring Program (NABat) mobile transect monitoring protocol. Blue lines depict 95% Bayesian credible intervals.

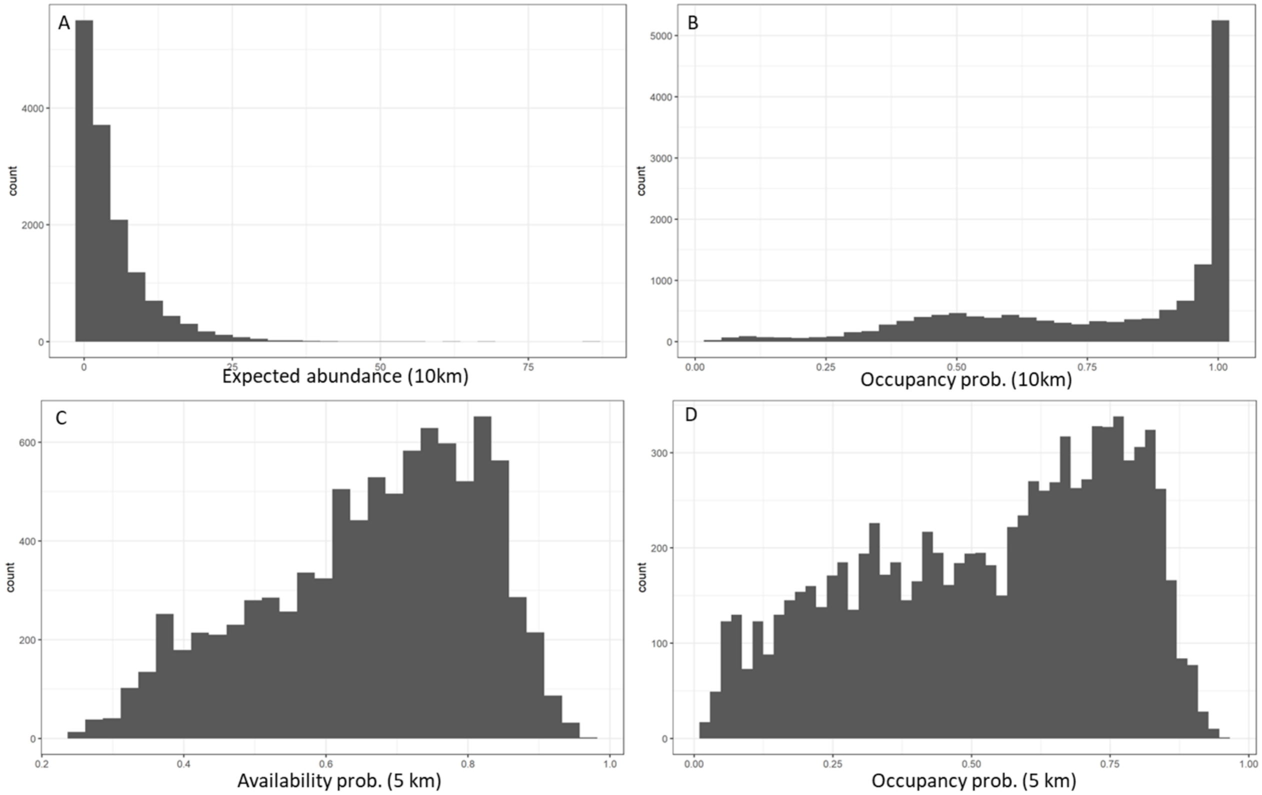

Supplementary Figure 6: Distribution of predicted values for expected abundance (10km) expected occupancy probability (10km), availability probability (5km) and total occupancy probability (5km) for all sampled locations for tricolored bats.

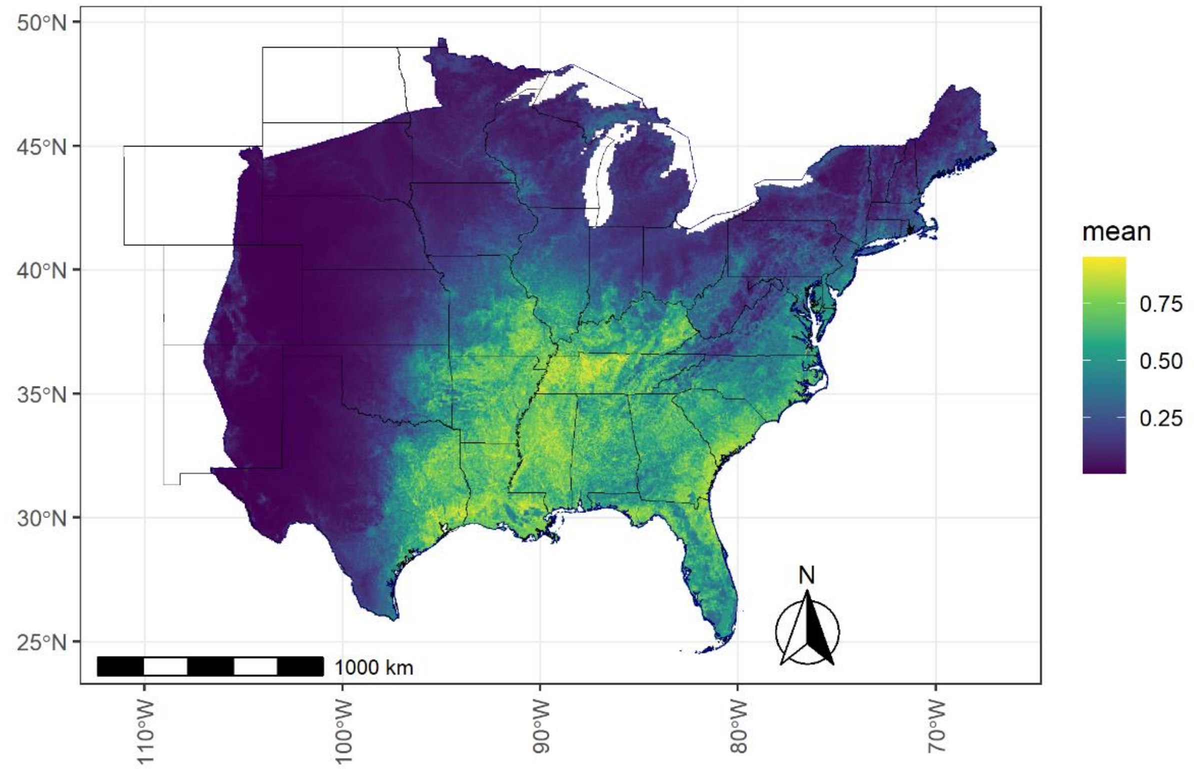

Supplementary Figure 7: Average occupancy probability (2017-2022 in the pre-volancy season) of tricolored bat in each North American Bat Monitoring Program (NABat) 5km grid cell quadrant across the modeled species range. This map corresponds with the USGS data release for the "Integrated Summer Species Distribution Model: Predicted Tricolored Bat Occupancy Probabilities. Version 1.1" (Udell et al. 2024)[17], which represents the prediction of the most current species distribution. State polygons are from the Database of Global Administrative Boundaries.

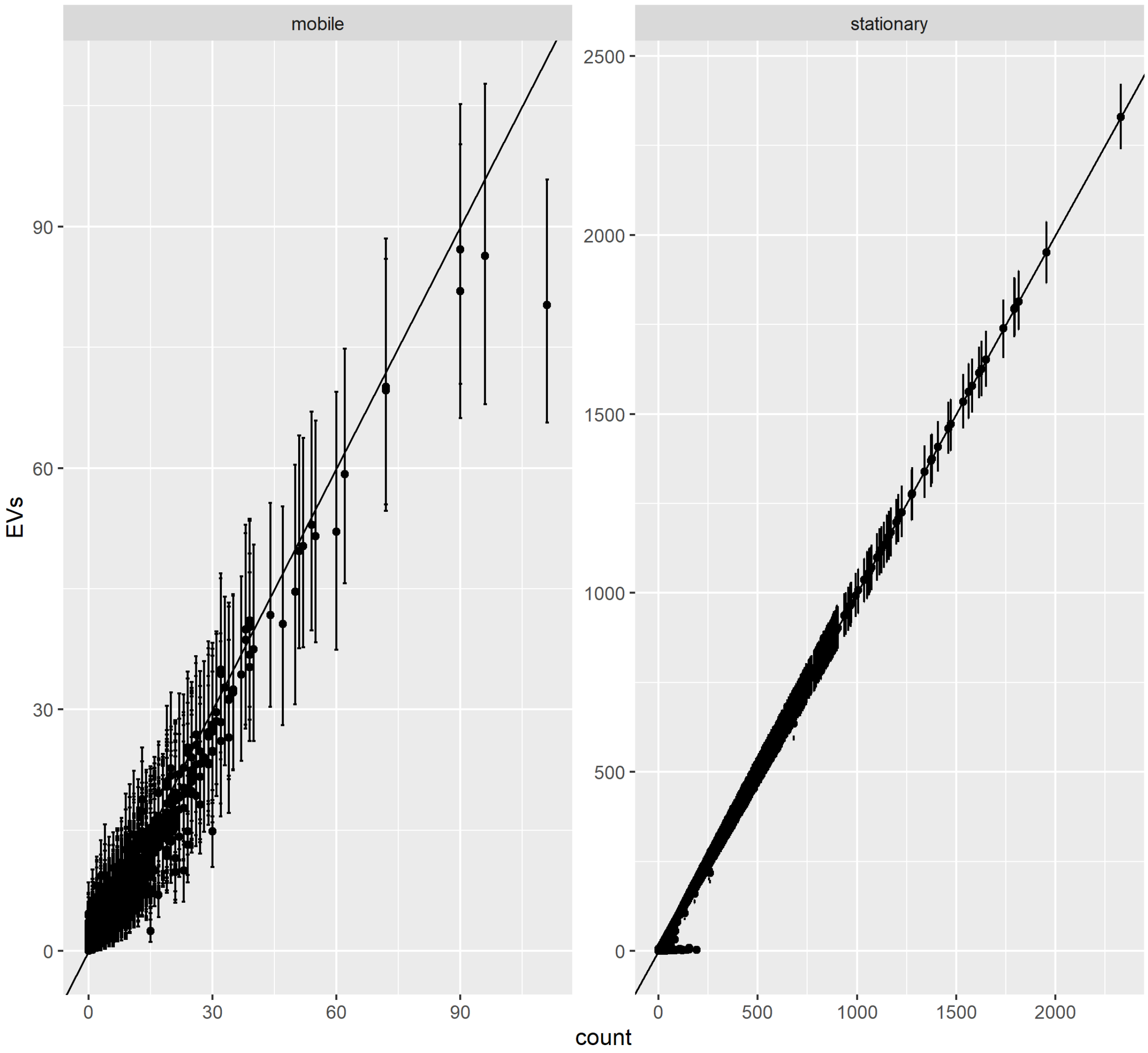

Supplementary Figure 8. A visual depiction of a posterior predictive check comparing the observed counts (x axis) to the expected values of counts (EVs, y axis) from mobile acoustics (left) to stationary acoustics (right). Error bars depict 95% Bayesian credible intervals.

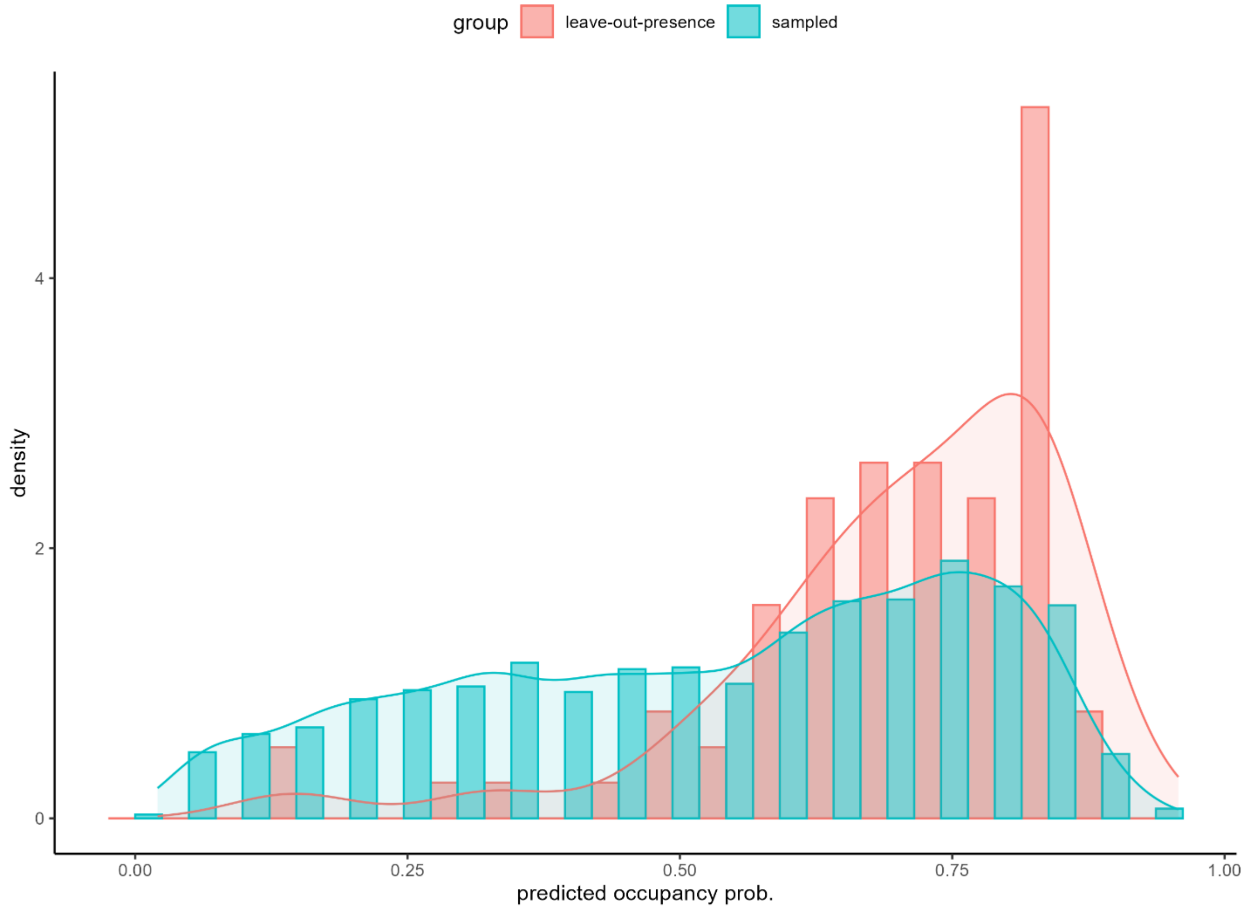

Supplementary Figure 9. Binned distributions of predicted unconditional occupancy probabilities (posterior means) for tricolored bats at the 5km quadrant scale for the leave-out group of confirmed presences (red, n=206) and for all sampled quadrants and years (blue, n=9036). The proportion of predicted occupancy probabilities for the leave-out group of confirmed presences were consistently higher in bins greater than 0.5, and consistently lower in bins < 0.5 compared to the background “all sampled” group.

Supplementary References

1. North American Bat Monitoring Program (NABat) Database v7.0.31 (Provisional Release): U.S. Geological Survey. Accessed 2023-05-24. NABat Request Number 166. https://doi.org/10.5066/P9UXA6CF

2. North American Bat Monitoring Program (NABat) Database v7.0.31 (Provisional Release): U.S. Geological Survey. Accessed 2023-05-24. NABat Request Number 167. https://doi.org/10.5066/P9UXA6CF

3. North American Bat Monitoring Program (NABat) Database v7.0.31 (Provisional Release): U.S. Geological Survey. Accessed 2023-05-24. NABat Request Number 172. https://doi.org/10.5066/P9UXA6CF

4. Global Administrative Areas (GADM). (2018, May 6). Version 3.6 [shapefiles]. University of California, Berkeley. Retrieved from [https://gadm.org/data.html]

5. Udell, B.J., Straw, B.R., Loeb, S.C., Irvine, K.M., Thogmartin, W.E., Lausen, C.L., Reichard, J.D., Coleman, J.T., Cryan, P.M., Frick, W.F. and Reichert, B.E., 2024. Using mobile acoustic monitoring and false-positive N-mixture models to estimate bat abundance and population trends. *Ecological Monographs*, *94*(4), p.e1617.

6. Doser, J.W., Finley, A.O., Weed, A.S. and Zipkin, E.F., 2021. Integrating automated acoustic vocalization data and point count surveys for estimation of bird abundance. *Methods in Ecology and Evolution*, *12*(6), pp.1040-1049.

7. Chambert, T., Grant, E.H.C., Miller, D.A., Nichols, J.D., Mulder, K.P. and Brand, A.B., 2018. Two-species occupancy modelling accounting for species misidentification and non-detection. *Methods in Ecology and Evolution*, *9*(6), pp.1468-1477.

8. Loeb, S.C., Rodhouse, T.J., Ellison, L.E., Lausen, C.L., Reichard, J.D., Irvine, K.M., Ingersoll, T.E., Coleman, J.T., Thogmartin, W.E., Sauer, J.R. and Francis, C.M., 2015. A plan for the North American bat monitoring program (NABat). *Gen. Tech. Rep. SRS-208. Asheville, NC: US Department of Agriculture Forest Service, Southern Research Station.*, *208*, pp.1-100.

9. Theobald, D.M., Harrison-Atlas, D., Monahan, W.B. and Albano, C.M., 2015. Ecologically-relevant maps of landforms and physiographic diversity for climate adaptation planning. *PloS one*, *10*(12), p.e0143619.

10. Wiens, A.M., Udell, B.J., Thogmartin, W.E., Straw, B.R., Cheng, T., Frick, W.F., Reichert, B.E. 2023. North American Bat Monitoring Program (NABat) Bayesian Hierarchical Model for Winter Abundance: Predicted Population Estimates (2022 and 2023). USGS data release. https://doi.org/10.5066/P9L0578M

11. Moilanen, A. and Hanski, I., 2001. On the use of connectivity measures in spatial ecology. Oikos, 95(1), pp.147-151.

12. Cheng, T.L., Reichard, J.D., Coleman, J.T., Weller, T.J., Thogmartin, W.E., Reichert, B.E., Bennett, A.B., Broders, H.G., Campbell, J., Etchison, K. and Feller, D.J., 2021. The scope and severity of white-nose syndrome on hibernating bats in North America. Conservation Biology, 35(5), pp.1586-1597.

13. Plummer, M., 2003, JAGS: a program for analysis of Bayesian graphical models using Gibbs sampling. In Proceedings of the 3rd international workshop on distributed statistical computing 124(125.10), pp. 1-10.

14. R Core Team. 2020. R: A language and environment for statistical computing. R Foundation

15. Kellner, K., and Meredith, M., 2019. Package ‘jagsUI’. A Wrapper Around 'rjags' to Streamline 'JAGS' Analyses. R Package Version, 1(1).

16. Gelman, A. and Rubin, D.B., 1992. Inference from iterative simulation using multiple sequences. Statistical Science 7(4), pp.457–511.

17. Udell, B.J., Stratton, C., Straw, B.R., Irvine, K.M., Reichard, J.D., Gaulke, S.M., Coleman, J.T.H., Tousley, F., Schuhmann, A.N., Inman, R.D., Shivley, R., Richert, B.E. 2023. North American Bat Monitoring Program (NABat) Integrated Summer Species Distribution Model: Predicted Tricolored Bat Occupancy Probabilities. Version 1.1. U.S. Geological Survey data release, https://doi.org/10.5066/P9MV37I7
