# Supplementary material for: Integrated distribution modeling resolves asynchrony between bat population impacts and occupancy trends through latent abundance
Source: Commun Biol. 2025 May 30;8:832. doi: 10.1038/s42003-025-08238-x (PMC12125192; doi:10.1038/s42003-025-08238-x)
Supplement: Supplementary file 2 — Reporting Summary [file 42003_2025_8238_MOESM2_ESM.pdf]

nature portfolio

Corresponding author(s): Bradley Udell

Last updated by author(s): May 5, 2025

Reporting Summary

Nature Portfolio wishes to improve the reproducibility of the work that we publish. This form provides structure for consistency and transparency in reporting. For further information on Nature Portfolio policies, see our Editorial Policies and the Editorial Policy Checklist.

Statistics

For all statistical analyses, confirm that the following items are present in the figure legend, table legend, main text, or Methods section.

n/a	Confirmed	
☐	☒	The exact sample size (*n*) for each experimental group/condition, given as a discrete number and unit of measurement
☐	☒	A statement on whether measurements were taken from distinct samples or whether the same sample was measured repeatedly
☐	☒	The statistical test(s) used AND whether they are one- or two-sided<br>*Only common tests should be described solely by name; describe more complex techniques in the Methods section.*
☐	☒	A description of all covariates tested
☐	☒	A description of any assumptions or corrections, such as tests of normality and adjustment for multiple comparisons
☐	☒	A full description of the statistical parameters including central tendency (e.g. means) or other basic estimates (e.g. regression coefficient) AND variation (e.g. standard deviation) or associated estimates of uncertainty (e.g. confidence intervals)
☐	☒	For null hypothesis testing, the test statistic (e.g. *F*, *t*, *r*) with confidence intervals, effect sizes, degrees of freedom and *P* value noted<br>*Give P values as exact values whenever suitable.*
☐	☒	For Bayesian analysis, information on the choice of priors and Markov chain Monte Carlo settings
☐	☒	For hierarchical and complex designs, identification of the appropriate level for tests and full reporting of outcomes
☐	☒	Estimates of effect sizes (e.g. Cohen's *d*, Pearson's *r*), indicating how they were calculated

Our web collection on statistics for biologists contains articles on many of the points above.

Software and code

Policy information about availability of computer code

Data collection	No software was used
Data analysis	We used program R version 4.1.2 and JAGS version 4.3. Annotated model code (JAGS) has been provided as supplemental material (Appendix S2) as additional documentation of model structure and priors, and as part of a USGS data release (https://doi.org/10.5066/P1FKYTMA)

For manuscripts utilizing custom algorithms or software that are central to the research but not yet described in published literature, software must be made available to editors and reviewers. We strongly encourage code deposition in a community repository (e.g. GitHub). See the Nature Portfolio guidelines for submitting code & software for further information.

Data

Policy information about availability of data

All manuscripts must include a data availability statement. This statement should provide the following information, where applicable:

- Accession codes, unique identifiers, or web links for publicly available datasets
- A description of any restrictions on data availability
- For clinical datasets or third party data, please ensure that the statement adheres to our policy

Data supporting this research are available from North American Bat Monitoring Program (NABat) database [https://sciencebase.usgs.gov/nabat/#/data/inventory], with restrictions [including non-disclosure agreements, licensing, other agreements]. The platform is developed and maintained by the USGS to provide shared, permission-controlled access to scientific data products and resources. Due to sensitivities around bat data including private land ownership and concern for the

safety of vulnerable populations, original data contributors are responsible for managing permissions and data access through the NABat Partner Portal. Users may restrict access to their project-level data, grant access upon request, or make data publicly available. Parties may request access to these data by following steps documented at https://www.nabatmonitoring.org/get-data. The parameters of the dataset drawn from the NABat database, date of the export and database version are documented in the citations and also available on the NABat Data Request Archive (see NABat Request Numbers 166, 167, 172) located at: https://sciencebase.usgs.gov/nabat/#/data/requests/all. Geospatial data used for modeling is available at: https://doi.org/10.5066/P9BPRLVL. Tabular versions of model outputs and predictions (including source data used to make figures) are available as an official USGS data release (https://doi.org/10.5066/P1FKYTMA).

Research involving human participants, their data, or biological material

Policy information about studies with human participants or human data. See also policy information about sex, gender (identity/presentation), and sexual orientation and race, ethnicity and racism.

Reporting on sex and gender	N/A
Reporting on race, ethnicity, or other socially relevant groupings	N/A
Population characteristics	N/A
Recruitment	N/A
Ethics oversight	N/A

Note that full information on the approval of the study protocol must also be provided in the manuscript.

Field-specific reporting

Please select the one below that is the best fit for your research. If you are not sure, read the appropriate sections before making your selection.

☐ Life sciences ☐ Behavioural & social sciences ☒ Ecological, evolutionary & environmental sciences

For a reference copy of the document with all sections, see nature.com/documents/nr-reporting-summary-flat.pdf

Ecological, evolutionary & environmental sciences study design

All studies must disclose on these points even when the disclosure is negative.

Study description	We used a multi-scale, integrated species distribution model to predict the relative abundance and occupancy probability (and trends over time in each) of tricolored bat across its continental range from 2012-2022 using data from multiple monitoring data streams (mobile acoustics, stationary acoustics, and live-capture) which were linked with the NABat master sampling grid. Monitoring protocols for each data stream are described in the methods and further in appendix S1, and Figure 1. Data were linked with the NABat master sampling grid at the 10km x 10km grid cell scale or 5km x 5km quadrant scale (depending on data type). Each data type contained temporal replication (multiple site nights per site) within each summer 'season' which is standard 'metapopulation design' that allows for estimating occupancy or abundance while accounting for imperfect detection (false-negatives). The number of observations for each monitoring type are documented in Table 1, and Appendix S2, Supplementary Table 1. Acoustic monitoring data consist of the number of 'bat-passes' (acoustic recordings) for the target species in each location and sampling night. Live captures consist of binary detection/non-detection summaries of bat-passes per night and sampled location. Observation models that appropriately accounted for false-positive detections were used for each acoustic data stream based on manual vetting or data preprocessing (e.g., the 'mle' decision rule to remove suspected false-positives). We used covariates at multiple spatial scales (grid cells and quadrants) to predict abundance at the grid-cell scale, and local availability at the quadrant scale, which together predict the overall occupancy probability at the quadrant scale. We also provide a comparison of inferences under the integrated model (in the trends of abundance and occupancy, in covariate effects, and in predictions of the species distribution) with each individual monitoring data stream.
Research sample	Samples are described in detail in the methods, and in Appendix S1. The research sample for all data types are correspond with bat populations in locations which have been monitored and for which data have been submitted to NABat, including a large portion of acoustic data collected under the NABat protocol (established in 2015). The NABat monitoring protocol stresses representative sampling to allowed for population level inferences across the range of the species. A map of depicting spatial sampling effort by monitoring type are provided in Appendix S1, Supplemental Figure 1.
Sampling strategy	Sampling strategy, protocols, and sampling sizes from a majority of the data contributors are following NABat protocols (e.g., 4 detectors per grid cell for 4 nights each) were established for NABat in "A plan for the North American Bat Monitoring Program" (Loeb et al. 2015) to allow for rigorous inferences across the continental range of bat species. Live-capture data is reported to NABat from a several different monitoring effects and protocols (statistically controlled for using data contributor level random effects). Data are accepted from all data contributors, include those following protocols which pre-date the NABat program.
Data collection	Data are collected by NABat Monitoring partners and data contributors (see methods for more details). As noted above, data

Data collection	collection procedures of the NABat protocol were established by Loeb et al. 2015, are further documented in the methods for each monitoring data type.
Timing and spatial scale	Data are from the summer season (May 1st - August 31st), the maternity season for bats, with two sub-seasons of interest (pre- July 15, before newborn pups of the year become volant, and after July 15th). Data span 2012- 2022. Relative abundance is estimated at the 10km x 10km grid cell scale, while occupancy is estimated at the grid cell and quadrant (5km x 5km) scale. The data are collected at the transect level (mobile transects) or at sampling points (stationary acoustic, live-capture).
Data exclusions	Data were excluded based on pre- established criteria, including: 1) the lack of geospatial information (coordinates) required to reconcile grid cell quadrants, 2) or due to reporting issues of some projects with manual vetting data (only the manual vetting data was excluded). For the stationary acoustic data: if a project had > 20 rows of confirmation data, and confirmation rates and review rates > 0.95, the vetting data was excluded for the entire project. This removed legacy and 'metadata' projects with improper reporting of manual review information. Manual vetting data was excluded from all projects suspected of "removal vetting" (i.e., those which only review 1 or 2 files to confirm a species presence instead of a larger subset of files, as identified as review rates < 0.05). For the mobile transect acoustic data, vetting data was censored from a single legacy project (project_id = 407) with known reporting issues.
Reproducibility	We have provided model code and described all analytical steps needed to reproduce the results.
Randomization	'GRTS' priority sampling is used in the NABat master sample to determine sampling priority of grid cells to promote spatial representativeness of sampling and allow for transferable inferences across the range of the species. We also used random effects (described in the methods) by strata (e.g., transect, project ID) to control for differences between data contributors and locations.
Blinding	Blinding was not relevant or possible for this study.

Did the study involve field work? ☒ Yes ☐ No

Field work, collection and transport

Field conditions	Field conditions were variable across the > 100,000 records analyzed in this work, but are documented using remote sensing information and daily weather summaries and used as predictive covariates of the species distribution, detection rates, and false-postie rates.
Location	The locations span the range of tricolored bat in the continental United States. All sampled locations are mapped in Appendix S1, Supplemental Figure 1.
Access & import/export	Habitats were accessed by each individual data contributor, following NABat protocols, or legacy monitoring protocols or state or federal agencies.
Disturbance	Acoustic sampling is designed to be minimally invasive. Mobile transects include some amount of car noise compared to stationary acoustic sampling, but are conducted along roads so as to not disturb habitat.

Reporting for specific materials, systems and methods

We require information from authors about some types of materials, experimental systems and methods used in many studies. Here, indicate whether each material, system or method listed is relevant to your study. If you are not sure if a list item applies to your research, read the appropriate section before selecting a response.

Materials & experimental systems

n/a	Involved in the study	
☒	☐	Antibodies
☒	☐	Eukaryotic cell lines
☒	☐	Palaeontology and archaeology
☒	☐	Animals and other organisms
☒	☐	Clinical data
☒	☐	Dual use research of concern
☒	☐	Plants

Methods

n/a	Involved in the study	
☒	☐	ChIP-seq
☒	☐	Flow cytometry
☒	☐	MRI-based neuroimaging

Plants

Seed stocks	NA
Novel plant genotypes	NA
Authentication	NA
